# Early-life oxidative stress programs persistent NFκB activation and neuroinflammation in autism spectrum disorder

**DOI:** 10.3389/fimmu.2026.1869130

**Published:** 2026-08-10

**Authors:** Yun Jiao, Qingzheng Jia, Xiaozhuang Zhang, Claire Zhang, Chongxia Wei, Dakota Wang, Xiaohan Liu, Huilin Li, Alex Hu, Liqin Zeng, Ling Li, Paul Yao

**Affiliations:** 1 Hainan Women and Children’s Medical Center, Hainan Medical University, Haikou, China; 2 Department of Veterinary and Animal Science, University of Massachusetts Amherst, Amherst, MA, United States; 3 Department of Engineering, Boston University, Boston, MA, United States; 4 Department of Gynecology, The Eighth Affiliated Hospital, Sun Yat-Sen University, Shenzhen, China

**Keywords:** autism spectrum disorder, developmental programming, neuroinflammation, NFκB, oxidative stress, redox signaling

## Abstract

**Background:**

Oxidative stress and immune dysregulation are hallmark features of autism spectrum disorder (ASD), yet whether oxidative imbalance acts as an upstream trigger of immune activation remains unclear. The redox-sensitive transcription factor NFκB represents a potential mechanistic link. We investigated whether early-life oxidative stress contributes to persistent NFκB activation and neuroinflammation in ASD.

**Methods:**

Umbilical cord blood and peripheral blood from ASD and typically developing children were analyzed for redox markers (GSH/GSSG ratio, malondialdehyde, 8-oxo-dG), NFκB activation (p65 DNA-binding and nuclear translocation), and inflammatory gene expression. The mechanistic relationship between oxidative stress and NFκB signaling was investigated in prenatal valproic acid (VPA)-exposed mice using antioxidant intervention (N-acetylcysteine, NAC), NFκB inhibition (Bay 11-7082), pro-oxidant challenge, behavioral assays, and primary amygdala neuron models.

**Results:**

ASD children exhibited persistent oxidative imbalance detectable at birth, accompanied by increased NFκB activation and pro-inflammatory gene expression. VPA-exposed mice recapitulated these molecular and behavioral abnormalities. In primary neurons, oxidative stress directly enhanced NFκB activity and promoter binding, whereas antioxidant and mitochondrial-targeted approaches suppressed NFκB activation. Developmentally, oxidative stress preceded sustained NFκB activation, and prenatal, but not postnatal, NAC treatment prevented these abnormalities. NAC restored redox homeostasis, reduced NFκB signaling, and improved behavioral deficits, whereas NFκB inhibition alone attenuated inflammatory responses but failed to correct oxidative imbalance or behavioral abnormalities.

**Conclusion:**

Early-life oxidative stress is an upstream pathogenic event associated with persistent NFκB activation and neuroinflammation in ASD. NFκB primarily mediates inflammatory signaling, while oxidative stress likely contributes to ASD-related behaviors through additional downstream pathways. These findings highlight early redox modulation as a potential therapeutic strategy.

## Introduction

Accumulating evidence has implicated oxidative stress as an important biological factor associated with autism spectrum disorder (ASD) (1, 2). When reactive oxygen species (ROS) production exceeds the capacity of antioxidant defense systems, oxidative imbalance can disrupt cellular signaling and promote oxidative damage (3, 4). Clinical and experimental studies have consistently reported increased oxidative damage to lipids, proteins, and DNA, together with impaired antioxidant defenses, in individuals with ASD and relevant animal models (5–9). In particular, the prenatal valproic acid (VPA) model reproduces oxidative stress and ASD-like neurodevelopmental abnormalities (10, 11). Our previous work further demonstrated reduced expression of the mitochondrial antioxidant enzyme superoxide dismutase 2 (SOD2) in rodent models of autism-like phenotypes, suggesting impaired mitochondrial redox regulation (12). Although oxidative stress is consistently associated with ASD, whether it functions as an upstream pathogenic driver or represents a secondary consequence of disease progression remains unclear.

ASD has also been associated with persistent immune dysregulation. Epidemiological studies indicate that maternal infection and prenatal inflammatory conditions modestly increase ASD risk (13), while analyses of ASD brain and peripheral blood have revealed elevated pro-inflammatory cytokines, activated microglia, and altered immune cell profiles (14). Likewise, maternal immune activation (MIA) models demonstrate that prenatal inflammatory signals can induce fetal neuroinflammation and ASD-like behaviors (15, 16). Nevertheless, it remains uncertain whether immune activation is a primary pathogenic event, a downstream consequence of oxidative stress, or part of a broader pathogenic network involving genetic, environmental, and metabolic factors (17, 18).

Among inflammatory signaling pathways, NFκB is a central regulator of innate immune responses and has been reported to be activated in individuals with ASD (19). Oxidative stress is a well-established upstream regulator of NFκB signaling through ROS-dependent activation of the IKK/IκB pathway (20, 21). However, whether oxidative stress directly drives persistent NFκB activation during ASD development has not been systematically investigated, and mechanistic studies combining antioxidant intervention with direct NFκB inhibition remain limited (22).

The amygdala is a key brain region involved in social behavior, emotional processing, and anxiety, all of which are frequently altered in ASD (23, 24). Experimental evidence suggests that the amygdala is particularly susceptible to oxidative stress and neuroinflammatory insults during neurodevelopment (9). Consistent with this concept, our previous studies demonstrated that prenatal environmental insults, including maternal diabetes and synthetic progestin exposure, induced ASD-like phenotypes accompanied by prominent molecular alterations in the amygdala (5, 6, 12). Therefore, the amygdala was selected as the primary brain region for mechanistic investigation in the present study.

In this study, we combined longitudinal human cohort analyses with a prenatal VPA mouse model to investigate the relationship between oxidative stress and NFκB activation in ASD. By integrating antioxidant (N-acetyl-L-cysteine) and NFκB inhibitor (BAY 11-7082) interventions with molecular, cellular, and behavioral analyses, we tested the hypothesis that oxidative stress acts upstream of NFκB activation and contributes to neuroinflammation associated with ASD.

## Materials and methods

**Supplementary Data S1** contains the detailed experimental procedures, while **Supplementary Table 1** summarizes the primer sequences used.

### Reagents and materials

The SOD2 antibody (sc-30080) was sourced from Santa Cruz Biotechnology. Antibodies against NFκB p65 (catalog #8242) and Histone H3 (catalog #9715) were purchased from Cell Signaling Technology, while the antibody for C-C motif chemokine ligand 3 (CCL3, #ab214819) was supplied by Abcam. Protein levels were quantified by the Coomassie Protein Assay Kit (Pierce Biotechnology). Nuclear and cytoplasmic protein fractions were prepared with the NE-PER Extraction Kit (Thermo Scientific, Cat. #78835, China). The antibody recognizing 8-oxo-dG (4354-MC-050) was acquired from Novus Biologicals. BAY 11-7082 (Cat. #196870), N-acetyl-L-cysteine (NAC, Cat. #A7250), and valproic acid (VPA, Cat. #P4543) were purchased from Sigma (China). NFκB p65 DNA-binding activity was evaluated using the TransAM^®^ NFκB p65 Chemi Kit (Active Motif, Cat. #40597) as per the supplier’s recommended procedure.

### Longitudinal birth cohort

Between July 2018 and July 2019, newborns delivered at Hainan Women and Children’s Medical Center (China) were prospectively enrolled. Umbilical cord blood (5–20 mL) was collected at delivery into EDTA-coated tubes, and umbilical cord blood mononuclear cells (UCBMCs) were separated. Total RNA preparation was conducted using Qiagen’s RNeasy Micro Kit and preserved for transcriptomic analyses, including RNA-sequencing and targeted assays. Clinical information and demographic variables such as parental age, perinatal conditions, and psychosocial traits were obtained using structured questionnaires. Developmental and behavioral assessments of enrolled children were performed at 6-month intervals until 3 years of age (July 2019–July 2022). Screening for autism was carried out using the Autism Behavior Checklist (ABC) and Autism Spectrum Quotient (AQ). Formal ASD diagnoses were confirmed by certified psychologists and psychiatrists according to DSM-5 criteria. Cognitive and adaptive performance was measured with the MSEL and VABS. Typically developing (TD) children were defined as those without ASD or developmental delays and with scores higher than 70 on both measures. Using these criteria, 29 participants were classified with ASD, while 31 age-matched TD controls were included (see **Supplementary Table 2**). UCBMCs from these participants were processed for RNA-seq analysis (see **Supplementary Figure 1a**) (7, 25). To validate the RNA-seq findings, an independent cohort comprising 41 ASD subjects and 41 age-matched TD controls was recruited from a separate study population (see **Supplementary Table 3**). Plasma and UCBMC samples from this validation cohort were collected for subsequent analyses (see **Supplementary Figure 1b**). Independent validation cohorts were used because the limited blood volume and sample consumption during RNA-seq precluded comprehensive downstream analyses on the same subjects.

### Population-based case-control cohort

A population-based study included children aged 2–6 years. ASD cases (n = 30) were identified through ABC screening and confirmed by DSM-5–based clinical evaluations. Typically developing (TD) controls (n = 28) were randomly selected from a larger pool of 2,519 TD children and matched to ASD participants by age, sex, and IQ (determined by WISC-IV). Control participants showed no evidence of ASD or developmental delay and had MSEL and VABS scores >70. Peripheral blood samples (3–5 mL) were collected at approximately 30 months of age (see **Supplementary Table 2**). PBMCs isolated from these participants were used for RNA-seq analysis to identify differentially expressed genes associated with ASD (see **Supplementary Figure 1a**) (7, 25). To validate the RNA-seq findings, an independent cohort comprising 56 ASD and 56 age-matched TD children from a separate study population was recruited (see **Supplementary Table 4**). PBMCs and plasma from this validation cohort were used for all subsequent analyses, including quantitative PCR, ChIP assays, 8-oxo-dG immunodetection, and oxidative stress measurements (GSH/GSSG ratio and MDA levels), but were not subjected to RNA-seq analysis (see **Supplementary Figure 1b**).

### RNA sequencing and bioinformatic analysis

Total RNA was isolated using TRIzol, and quality was assessed by NanoDrop and Agilent Bioanalyzer. Libraries were prepared with the VAHTS Universal V6 kit and sequenced on an Illumina NovaSeq 6000 platform (150-bp paired-end). Raw reads were quality-filtered with fastp and aligned to the reference genome using HISAT2. Gene counts were generated with HTSeq, and expression levels were calculated as FPKM. Differential expression was identified using DESeq2 (|log_2_ fold change| > 1, adjusted p < 0.05). Sample variability was assessed by PCA and hierarchical clustering in R (v3.2.0). To investigate functional relevance, enrichment testing was applied to identify overrepresented terms in GO, KEGG, Reactome, and WikiPathways, using hypergeometric probability models. Broader pathway-level alterations were explored with Gene Set Enrichment Analysis (GSEA) software. Additional analyses included transcript reconstruction with StringTie, assessment of alternative splicing using ASprofile, discovery of single-nucleotide variants and small insertions/deletions via SAMtools and BCFtools, and annotation of variant effects with SnpEff (26–31).

### Oxidative stress evaluation

Reactive oxygen species (ROS) formation was assessed with CM-H2DCFDA (10 µM; Invitrogen), incubated at 37 °C for 45min, and fluorescence signals were determined at 485/530nm and quantified as nmol/mL using an H2O2 calibration curve. Oxidative DNA damage was assessed by immunostaining for 8-oxo-7,8-dihydro-2′-deoxyguanosine (8-oxo-dG), a marker of oxidative modification of guanine residues in DNA. The staining intensity was quantified using ImageJ software. Circulating oxidative DNA damage levels were evaluated by measuring plasma 8-hydroxy-2′-deoxyguanosine (8-OHdG) concentrations using a human 8-OHdG ELISA kit (Cloud-Clone Corp, CEA660Ge, Wuhan, China), according to the manufacturer’s instructions. The GSH/GSSG ratio, indicating glutathione redox status, was determined by the GSH/GSSG-Glo™ Assay Kit (Promega, V6611) as per supplier’s protocol.

### Malondialdehyde quantification

MDA levels in plasma and tissues were measured using a commercial ELISA kit (Nanjing Jiancheng). Plasma samples were centrifuged, and tissues were homogenized in PBS and clarified by centrifugation. Supernatants were stored at −80 °C until analysis. Assays were performed according to the manufacturer’s protocol, with absorbance read at 450 nm. MDA concentrations were expressed as nmol/mL (plasma) or nmol/mg protein (tissues).

### Immunostaining

Cells on gelatin-coated coverslips were fixed, permeabilized, and incubated with antibodies against CCL3, NFκB p65, or 8-oxo-dG. After washes, fluorophore-labeled secondary antibodies (FITC or Texas Red) were applied, and nuclei were counterstained with DAPI. Samples were mounted and imaged, and fluorescence intensity was quantified using ImageJ.

### *In vivo* animal experiments

Ten timed-pregnant dams were randomly assigned to each prenatal treatment group before VPA or saline administration. To minimize litter effects and ensure statistical independence, one male offspring was randomly selected from each litter for subsequent experiments. To account for perinatal mortality, a minimum of nine viable male pups were included per group. Selected offspring were housed individually in separate cages to minimize potential maternal or social influences. Investigators involved in behavioral testing, histological evaluation, and data analysis were blinded to treatment allocation to minimize potential bias.

### Postnatal treatment in prenatal VPA-exposed offspring

Timed-pregnant C57BL/6 mice received a single intraperitoneal injection of VPA (600 mg/kg in saline) on gestational day 12.5, while control dams received saline. Male offspring were randomly assigned into four groups: CTL/VEH (prenatal saline + vehicle), VPA/VEH (prenatal VPA + vehicle), VPA/NAC (prenatal VPA + NAC, 150 mg/kg), and VPA/Bay11-7082 (prenatal VPA + Bay 11-7082, 5 mg/kg). The doses of NAC and Bay 11–7082 were selected based on previous studies demonstrating effective modulation of oxidative stress and NF-κB signaling in rodent models (32, 33). From postnatal day (PND) 21 to 41, mice received daily intraperitoneal injections of the assigned treatments. This period was selected because PND 21–41 represents a juvenile-to-adolescent developmental window in mice, during which neural maturation, immune regulation, and behaviorally relevant developmental processes remain active (34). Neonatal PBMCs were isolated at PND 1–5. Behavioral testing occurred at PND 42–49. On PND 50, mice were euthanized; blood, plasma, and PBMCs were collected. Brain tissues were dissected, snap-frozen for molecular analyses, or fixed for immunohistochemistry. Gene expression was measured by qPCR, NFκB pathway activation by western blot, and plasma proinflammatory cytokines by ELISA. Oxidative stress markers -including ROS, MDA, and GSH/GSSG ratio -were quantified to assess redox imbalance (see **Supplementary Figure 2**). In addition, all animals were included in behavioral assessments (n=9/group); however, due to limited tissue availability and the need to allocate specimens across multiple molecular and biochemical assays, a subset of 5 animals per group was used for specific biological analyses.

### Animal behavior assessment

Behavioral assessments were conducted in mouse offspring using the Marble-Burying Test (MBT) to evaluate repetitive behavior, the Open-Field Test (OFT) to determine anxiety-like behavior, and the Three-Chambered Social Test to measure sociability and social novelty preference (6, 12, 35–37).

### Preparation of PBMC and plasma from neonatal mice via decapitation

Neonatal mice (postnatal day 1–5) were euthanized by rapid decapitation with sterile surgical scissors. Immediately following decapitation, the body was held vertically over a sterile 1.5 mL low-retention microcentrifuge tube preloaded with 2–5 µL of anticoagulant (0.5M EDTA) to prevent coagulation. Blood was allowed to drain passively from the neck, and gentle massage from tail to neck was applied to maximize yield. Samples were kept on ice and processed within 1 h. Plasma was isolated by centrifugation (2,000×g, 10 min, 4 °C) and stored at −80 °C in RNase-/DNase-free tubes. The remaining cellular fraction was resuspended in 500µL PBS, washed at 500×g for 5min at 4 °C, and the isolated PBMC pellet was immediately used for the analyses of gene expression, oxidative stress, and NFκB p65 activation. Typical yields ranged from 50-100µL of whole blood per pup, providing approximately 20–50 µL of plasma and sufficient cells for molecular analysis.

### Isolation of brain tissues

Experimental mice were deeply anesthetized with isoflurane vapor (>5% via free breathing). Blood was collected via cardiac puncture for PBMC isolation, followed by transcardial perfusion with 20 mL ice-cold perfusion solution over 5 min. Brains were gently extracted and submerged in ice-cold DPBS. Using a surgical microscope and The Mouse Brain in Stereotaxic Coordinates (3rd Edition) as a reference, target regions -including the amygdala, hypothalamic paraventricular nucleus (PVN), and hippocampus -were dissected. Each structure was collected into separate dishes, with all dissections completed within one hour. Tissues were promptly frozen at −80 °C for molecular assays or future experiments (38, 39).

### Immunohistochemistry

Frozen brain sections (10μm) were fixed in 2% paraformaldehyde, methanol-treated, permeabilized, and incubated with anti-SOD2 antibody, followed by Texas Red–conjugated secondary antibody. Nuclei were counterstained with DAPI in a fluorescent mounting medium, and coverslips were applied. Fluorescence microscopy was used to capture images, and SOD2 expression was quantified in 60 cells per experimental group using ImageJ software.

### Proinflammatory cytokine measurement by ELISA

Plasma levels of BCL2-related protein A1 (BCL2A1), CCL3, G0/G1 switch 2 (G0S2), interleukin-1 beta (IL1β) and tumor necrosis factor alpha (TNFα) were quantified in human and mouse samples using species-specific ELISA kits (R&D Systems, China). Samples were centrifuged and kept at −80 °C until analysis. Following the kit instructions, standards and appropriately diluted samples were added to 96-well plates coated with capture antibodies and incubated at 37 °C for 2h. Biotinylated detection antibodies, HRP-streptavidin, and TMB substrate were subsequently applied. Reactions were stopped with 2 N H2SO4, absorbance was read at 450 nm, and cytokine levels were calculated from standard curves, with all samples measured in duplicate, and human and mouse assays were performed in parallel with matched antibody pairs.

### Temporal and mechanistic validation of oxidative stress–driven NFκB activation

For the developmental time-course analysis, amygdala tissues from VPA-exposed and control offspring were collected at embryonic day 18.5 (E18.5), postnatal day 7 (P7) or 21 (P21), and analyzed for redox indices (GSH/GSSG ratio and MDA), NFκB activation, and inflammatory gene expression by RT-qPCR (see **Supplementary Figure 3a**). To assess sufficiency, wild-type P7 pups were acutely treated with pro-oxidants (rotenone or menadione) and examined 24–48 h later for ROS generation and NFκB activation. To evaluate the temporal requirement for antioxidant intervention, NAC was administered either prenatally to dams (E10.5-E18.5) or postnatally to pups (P7-P21), followed by assessment of redox status and NFκB signaling (see **Supplementary Figure 3b**). Primary amygdala neurons were used to interrogate cellular mechanisms. Hydrogen peroxide (H2O2) induced ROS production and NFκB p65 activation in a dose-dependent manner, whereas NAC treatment or SOD2 overexpression (↑SOD2) effectively attenuated these responses. ChIP assays were performed to quantify p65 occupancy at the IL1β and TNFα promoters (see **Supplementary Figure 3c**). All experiments included appropriate vehicle controls and technical standards, and analyses were performed in a blinded manner.

## Results

### Sixteen downstream NFκB target genes were upregulated in both umbilical cord and peripheral blood of ASD subjects

**Figure 1:**
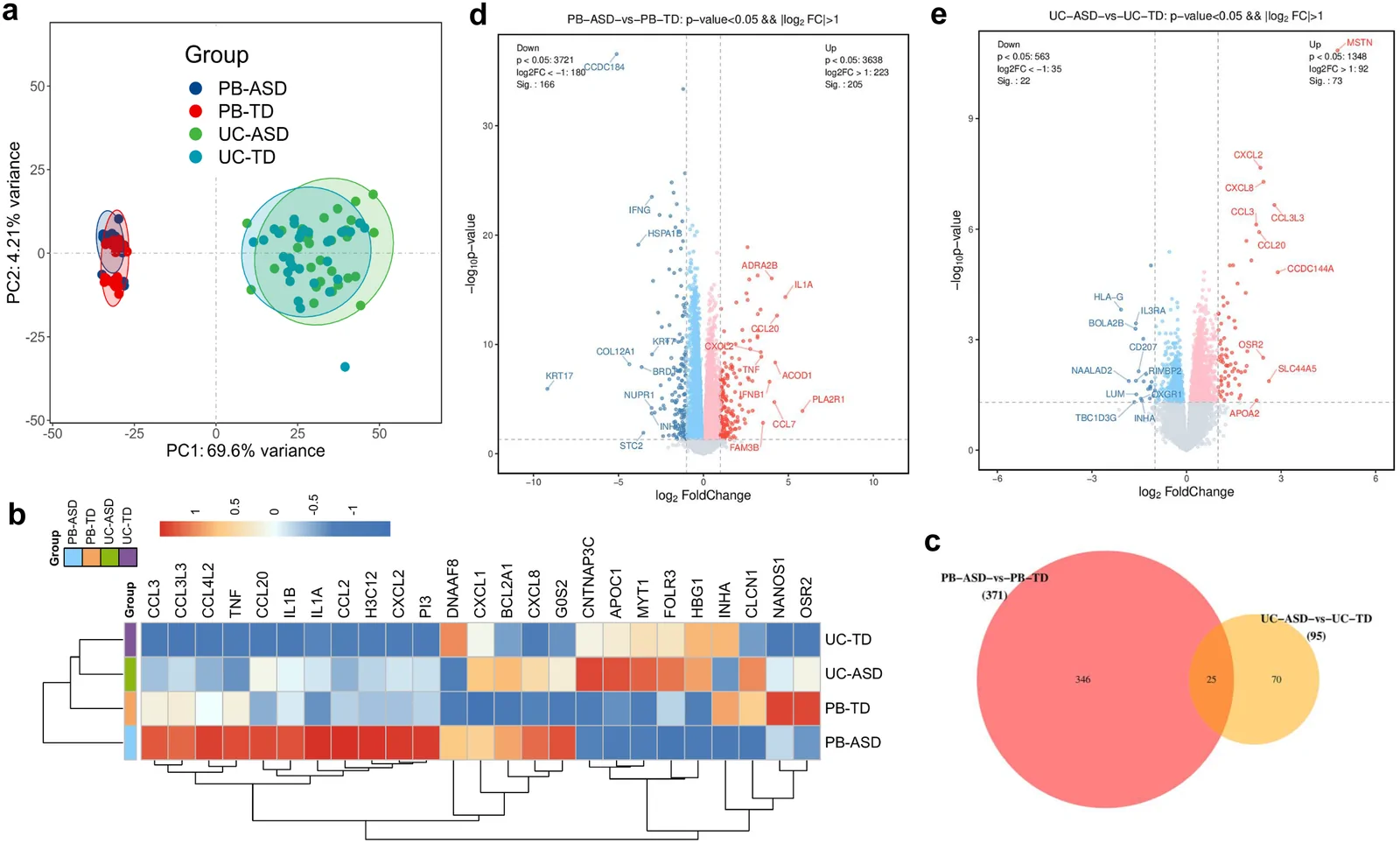
Sixteen NFκB downstream genes are upregulated in umbilical cord (UC) and peripheral blood (PB) of ASD subjects. UC and PB samples from typically developing (TD) and ASD subjects were analyzed by RNA-Seq. **(a)** PCA plot showing group separation. **(b)** Heatmap of 25 overlapping differentially expressed genes. **(c)** Venn diagram of shared and unique differentially expressed genes. **(d, e)** Volcano plots of differentially expressed genes in PB **(d)** and UC **(e)**. Gray dots represent non-significant genes, red and blue dots indicate significant up- and down-regulated genes, respectively. The x-axis shows log_2_(fold change), and the y-axis shows -log_10_(p-value). The individual group sizes are as follows: UC-TD (n = 31), UC-ASD (n = 29), PB-TD (n = 28), and PB-ASD (n = 30).

UC and PB samples were collected from ASD and typically developing (TD) individuals, forming four groups for RNA-Seq: UC-ASD (n=29), UC-TD (n=31), PB-ASD (n=30), and PB-TD (n=28). PCA revealed clear separation between UC and PB along PC1 (69.6% variance), with UC samples tightly clustered and PB samples more dispersed, indicating higher variability in PB gene expression. Within each blood type, ASD and TD subjects did not form distinct subclusters, suggesting that blood source rather than diagnosis primarily drives transcriptomic differences (**Figure 1a**). Heatmaps and hierarchical clustering highlighted blood source–specific expression patterns (**Figure 1b**). PB-ASD samples showed pronounced upregulation of inflammatory and chemokine genes (e.g., CCL3, CCL4L2, IL1A, CXCL8) compared to PB-TD, whereas UC samples exhibited fewer changes between ASD and TD. Venn analysis revealed 371 DEGs in PB-ASD versus PB-TD and 95 in UC-ASD versus UC-TD, with only 25 genes overlapping, indicating largely distinct transcriptional alterations between blood sources (**Figure 1c**). Volcano plots further confirmed these differences, with 205 genes upregulated and 166 downregulated in PB-ASD, including increased IL1A, TNF, CCL20, CXCL2, and decreased IFNG, HSPA1B, COL12A1. In UC-ASD, 73 genes were upregulated (e.g., CCL3, CCL3L3, CCL20, CXCL2, CXCL8) and 22 downregulated (e.g., HLA-G, INHA) (**Figures 1d, e**). Of the 25 overlapping DEGs, 16 were upregulated in both UC and PB of ASD subjects, most regulated directly or indirectly by NFκB. After filtering for expression >2.0 and ASD/TD ratio >1.6, five NFκB-linked genes were selected for further analysis: BCL2A1, CCL3, G0S2, IL1β, and TNFα (**Table 1**).

**Table 1:** Expression trends of 25 overlapped genes between UC and PB groups.

Gene ID	UC-ASD (n=29)	UC-TD (n=31)	UC-ASD/ UC-TD ratio	Up/Down regulation	PB-ASD (n=30)	PB-TD (n=28)	PB-ASD/ PB-TD ratio	Up/Down regulation
CCL3	41.5966	10.9086	3.81	Up	694.6820	109.8827	6.32	Up
CCL3L3	28.4757	4.7575	5.99	Up	281.3252	59.0949	4.76	Up
CCL4L2	4.7177	1.5504	3.04	Up	95.6229	10.2986	9.29	Up
TNF	5.6607	2.6822	2.11	Up	373.7509	34.7910	10.74	Up
CCL20	4.5017	0.1521	29.59	Up	28.4590	1.4130	20.14	Up
IL1B	81.2366	12.2342	6.64	Up	771.3689	72.9410	10.58	Up
IL1A	0.3524	0.0254	13.86	Up	1.9518	0.0676	28.85	Up
CCL2	0.3836	0.2240	1.71	Up	1.7862	0.4734	3.77	Up
H3C12	0.1712	0.0910	1.88	Up	0.4194	0.1573	2.67	Up
CXCL2	3.5879	0.8292	4.33	Up	27.5362	2.6446	10.41	Up
PI3	0.5730	0.3117	1.84	Up	1.2393	0.5430	2.28	Up
DNAAF8	0.0103	0.0247	0.42	Down	0.0221	0.0091	2.44	Up
CXCL1	5.6037	3.4173	1.64	Up	5.2394	0.5473	9.57	Up
BCL2A1	148.7299	90.0907	1.65	Up	154.8526	71.7550	2.16	Up
CXCL8	141.5085	32.2367	4.39	Up	284.7329	41.2422	6.90	Up
G0S2	158.6259	82.9900	1.91	Up	351.1935	65.8745	5.33	Up
CNTNAP3C	0.2855	0.1232	2.32	Up	0.0153	0.0049	3.09	Up
APOC1	0.6593	0.3481	1.89	Up	0.0663	0.1309	0.51	Down
MYT1	0.6297	0.3486	1.81	Up	0.0036	0.0101	0.35	Down
FOLR3	53.6622	26.1056	2.06	Up	5.2164	14.5946	0.36	Down
HBG1	4198.9375	2746.1816	1.53	Up	5.7852	11.6704	0.50	Down
INHA	0.0154	0.0449	0.34	Down	0.0054	0.0470	0.12	Down
CLCN1	0.0529	0.0277	1.91	Up	0.0193	0.0466	0.41	Down
NANOS1	0.0162	0.0082	1.98	Up	0.0151	0.0285	0.53	Down
OSR2	0.0261	0.0051	5.15	Up	0.0110	0.0508	0.22	Down

The expression number in the above table denotes a DESeq2-normalized count threshold applied during gene filtering and differential expression analysis.

### Elevated NFκB activation and oxidative stress in umbilical cord blood of ASD subjects

**Figure 2:**
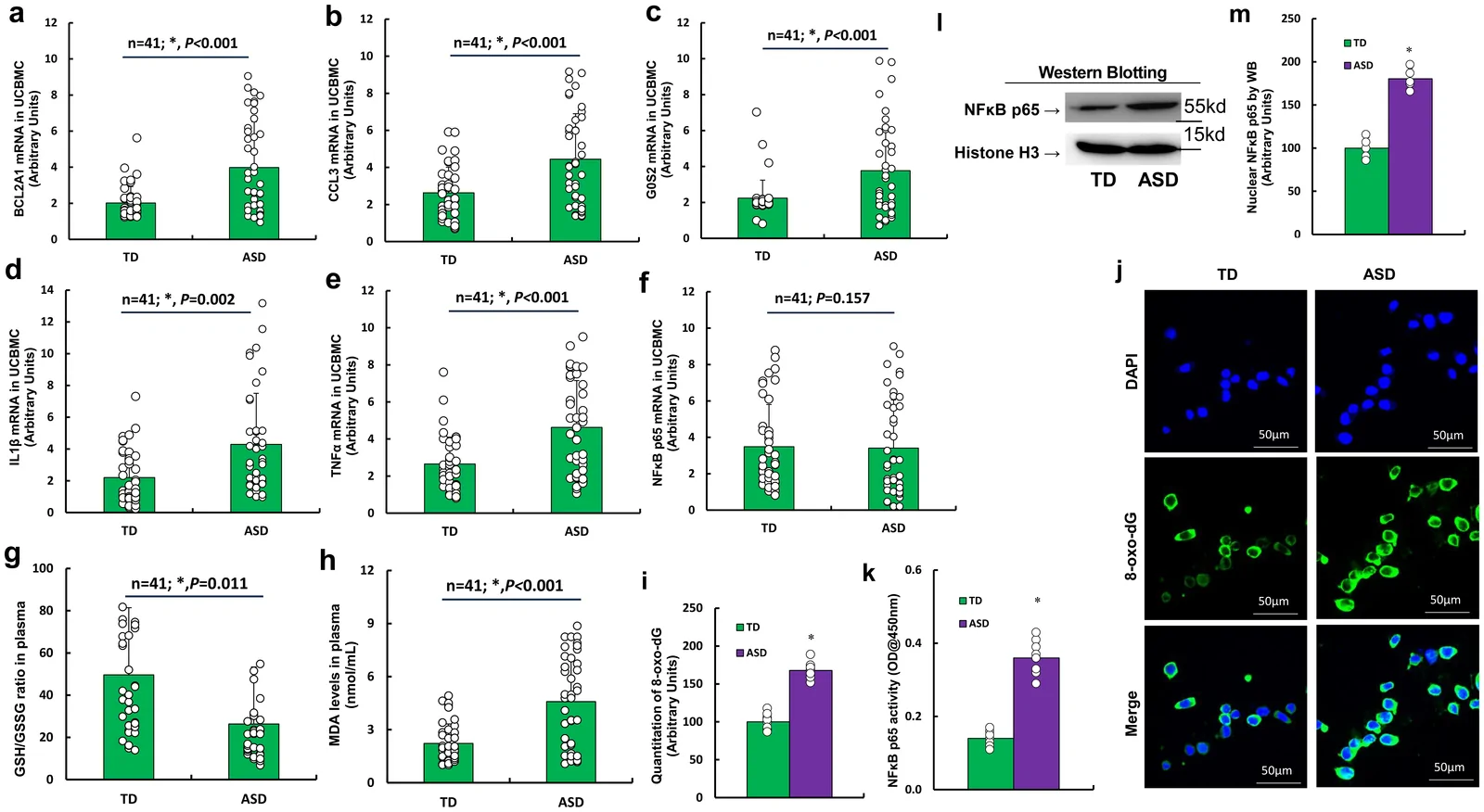
Increased NFκB pathway activation and oxidative stress in umbilical cord blood of ASD subjects. UCBMC and plasma were isolated from UC blood of TD and ASD subjects (n=41). **(a–f)** mRNA levels by qRT-PCR for BCL2A1, CCL3, G0S2, IL1β, TNFα, and NFκB p65 in PBMC, n=41. **(g-i)** Oxidative stress markers: GSH/GSSG ratio in plasma **(g)**, n=41; plasma MDA **(h)**, n=41; and PBMC 8-oxo-dG levels **(i)**, n=7. **(j)** Representative images for **(i)**. **(k)** NFκB p65 activity, n=7. **(l)** Representative western blot of nuclear NFκB p65. **(m)** Quantification of **(l)**, n=7. *P<0.05 vs. TD. Data are mean ± SEM. For the last 3 parameters, 7 samples per group were selected because these assays required more specimen, which was not available for all 41 subjects.

PBMCs and plasma from umbilical cord blood of TD and ASD subjects (n=41) were analyzed. mRNA levels of BCL2A1, CCL3, G0S2, IL1β, and TNFα were significantly higher in ASD subjects, while total NFκB p65 expression was unchanged (**Figures 2a–f**). Redox analysis revealed decreased GSH/GSSG ratio and increased plasma MDA, alongside elevated 8-oxo-dG in PBMC of ASD subjects (**Figures 2g–j**). NFκB pathway activation was confirmed by increased p65 activity and enhanced nuclear translocation in ASD PBMC compared with TD controls (**Figures 2k–m**; **Supplementary Figure 4a**).

### Elevated NFκB activation and oxidative stress in peripheral blood of ASD subjects

**Figure 3:**
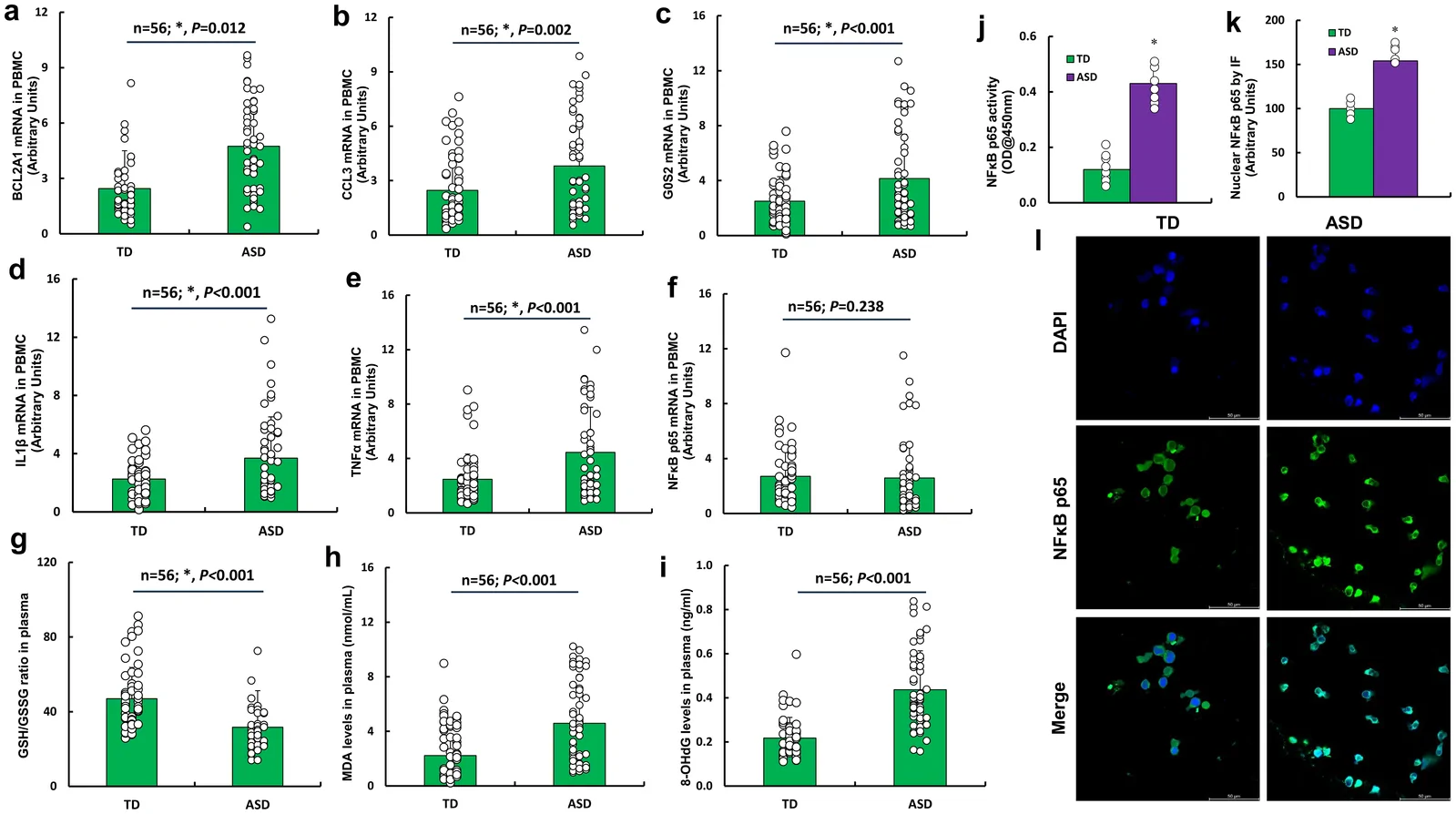
Increased NFκB pathway activation and oxidative stress in peripheral blood of ASD subjects. PBMC and plasma were collected from TD and ASD subjects (n=56). **(a–f)** mRNA levels by qRT-PCR for BCL2A1, CCL3, G0S2, IL1β, TNFα, and NFκB p65, n=56. **(g–i)** Oxidative stress markers: plasma GSH/GSSG ratio **(g)**, MDA **(h)**, and 8-OHdG **(i)**, n=56. **(j)** NFκB p65 activity, n=7. **(k)** Nuclear NFκB p65 quantification, n=7. **(l)** Representative nuclear NFκB p65 images for **(k)**. *P<0.05 vs. TD. Data are mean ± SEM. Note: For the last 2 parameters, 7 samples per group were selected because these assays required more specimen, which was not available for all 56 subjects.

PBMC and plasma from TD and ASD subjects (n=56) were analyzed. mRNA levels of BCL2A1, CCL3, G0S2, IL1β, and TNFα were significantly higher in ASD, while total NFκB p65 expression was unchanged (**Figures 3a–f**). Redox analysis revealed decreased GSH/GSSG ratio and increased plasma MDA and 8-OHdG in ASD subjects (**Figures 3g–i**). NFκB activation was confirmed by elevated p65 activity and enhanced nuclear translocation in ASD PBMC compared with TD controls (**Figures 3j–l**).

### Prenatal VPA exposure triggers NFκB activation and oxidative stress in neonatal offspring

**Figure 4:**
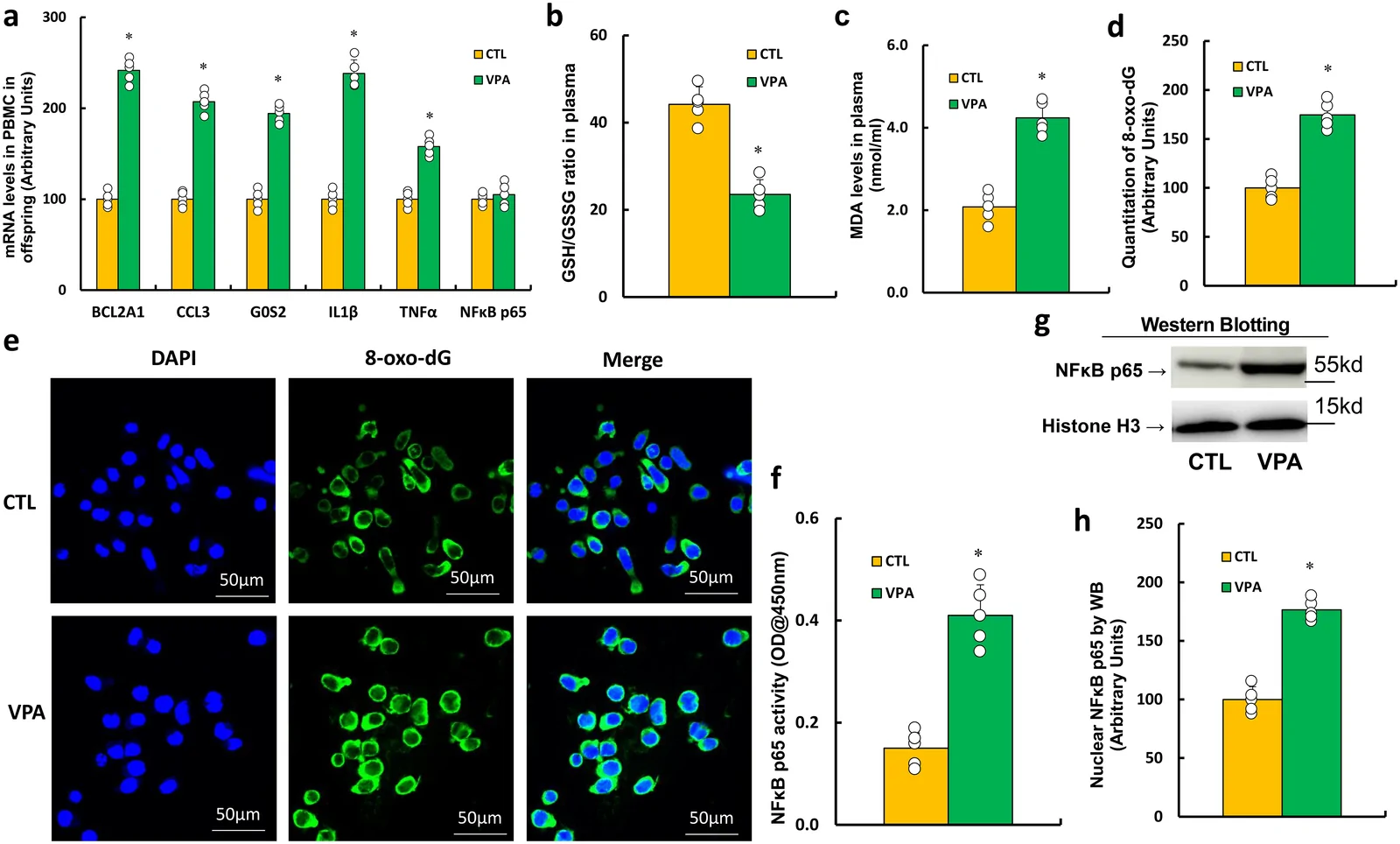
Prenatal VPA exposure induces NFκB activation and oxidative stress in neonatal offspring. Pregnant dams were injected with vehicle (CTL) or VPA. PBMC and plasma were collected from neonatal offspring. **(a)** mRNA expression in PBMC. **(b)** Plasma GSH/GSSG ratio. **(c)** Plasma MDA levels. **(d)** PBMC 8-oxo-dG formation. **(e)** Representative images for **(d)**. **(f)** NFκB p65 activity. **(g)** Representative nuclear NFκB p65 western blot. **(h)** Quantification of **(g)**. n=5. *P<0.05 vs. CTL. Data are mean ± SEM.

PBMC and plasma from neonatal offspring of control (CTL) or VPA-exposed dams were analyzed. ASD offspring exhibited significantly higher mRNA levels of BCL2A1, CCL3, G0S2, IL1β, and TNFα, while total NFκB p65 remained unchanged (**Figure 4a**). Redox analysis showed decreased plasma GSH/GSSG ratio, increased MDA, and elevated 8-oxo-dG in PBMC (**Figures 4b–e**). NFκB pathway activation was confirmed by increased p65 activity and nuclear translocation in ASD PBMCs (**Figures 4f–h**; **Supplementary Figure 4b**).

### Antioxidant NAC and NFκB inhibitor Bay 11–7082 partially reverse prenatal VPA-induced NFκB activation

**Figure 5:**
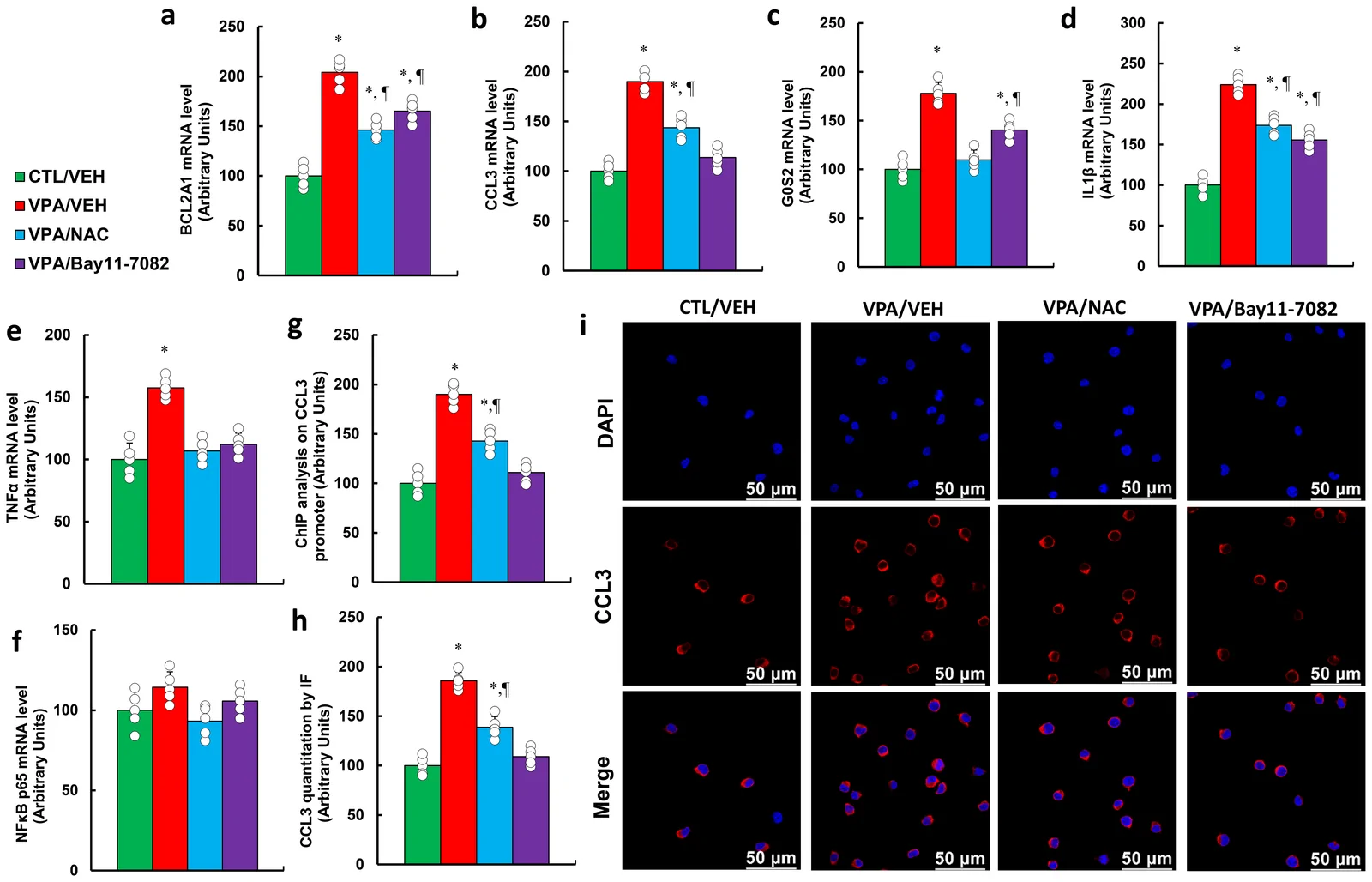
Prenatal VPA-induced NFκB pathway activation is partially rescued by antioxidant NAC and the NFκB inhibitor Bay 11-7082. Male offspring from control (CTL) or VPA-treated dams received injections of vehicle (VEH), NAC, or Bay 11-7082. PBMC were collected for analysis. **(a–f)** qPCR quantification of BCL2A1 **(a)**, CCL3 **(b)**, G0S2 **(c)**, IL1β **(d)**, TNFα **(e)**, and NFκB p65 **(f)**. **(g)** ChIP assay of NFκB p65 binding to the CCL3 promoter. **(h)** CCL3 protein levels by immunostaining. **(i)** Representative images for **(h)**. n=5. *P<0.05 vs. CTL/VEH; ^¶^P<0.05 vs. VPA/VEH. Data are mean ± SEM.

Male offspring from control (CTL) or VPA-treated dams received vehicle (VEH), NAC (150 mg/kg), or Bay 11-7082 (5 mg/kg), and PBMC were analyzed. VPA/VEH significantly increased BCL2A1, CCL3, G0S2, IL1β, and TNFα mRNA, while total NFκB p65 remained unchanged (**Figures 5a–f**). NAC or Bay 11–7082 partially or fully reversed these increases. ChIP showed enhanced NFκB p65 binding to the CCL3 promoter in VPA/VEH, partially blocked by NAC and fully by Bay 11-7082 (**Figure 5g**). Immunostaining of CCL3 mirrored the mRNA results (**Figures 5h, i**).

### Prenatal VPA-induced oxidative stress and NFκB activation are fully reversed by NAC and partially by Bay 11-7082

**Figure 6:**
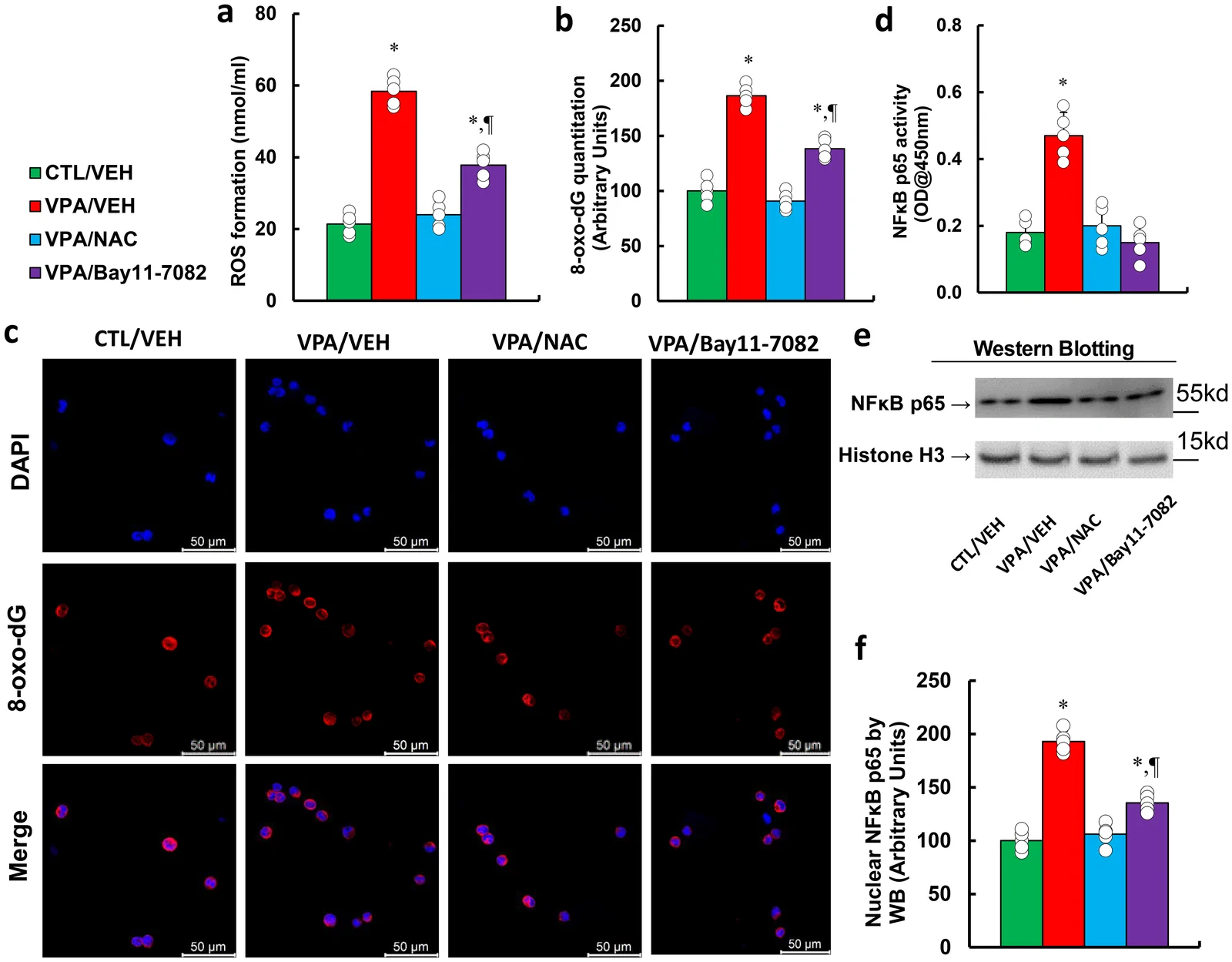
Prenatal VPA-induced oxidative stress and NFκB activation is fully rescued by antioxidant NAC, while Bay 11–7082 has partial effects. Male offspring from CTL or VPA-treated dams received VEH, NAC, or Bay 11-7082. PBMC and plasma were analyzed. **(a)** ROS formation in PBMC. **(b)** PBMC 8-oxo-dG levels. **(c)** Representative images for **(b)**. **(d)** NFκB p65 activity. **(e)** Representative nuclear NFκB p65 western blot. **(f)** Quantification of **(e)**. n=5. *P<0.05 vs. CTL/VEH; ^¶^P<0.05 vs. VPA/VEH. Data are mean ± SEM.

Male offspring from control (CTL) or VPA-treated dams received VEH, NAC, or Bay 11-7082, and PBMC and plasma were analyzed. VPA/VEH increased ROS, decreased GSH/GSSG ratio, and elevated 8-oxo-dG in PBMC (**Figures 6a–c**), effects fully reversed by NAC and partially by Bay 11-7082. VPA/VEH also enhanced NFκB p65 activity and nuclear translocation (**Figures 6d–f**, **S4c**), fully reversed by NAC and partially by Bay 11-7082.

### Prenatal VPA exposure induced autism-like behaviors that were partially ameliorated by the antioxidant NAC, whereas the NFκB inhibitor Bay 11–7082 showed minimal behavioral benefit

**Figure 7:**
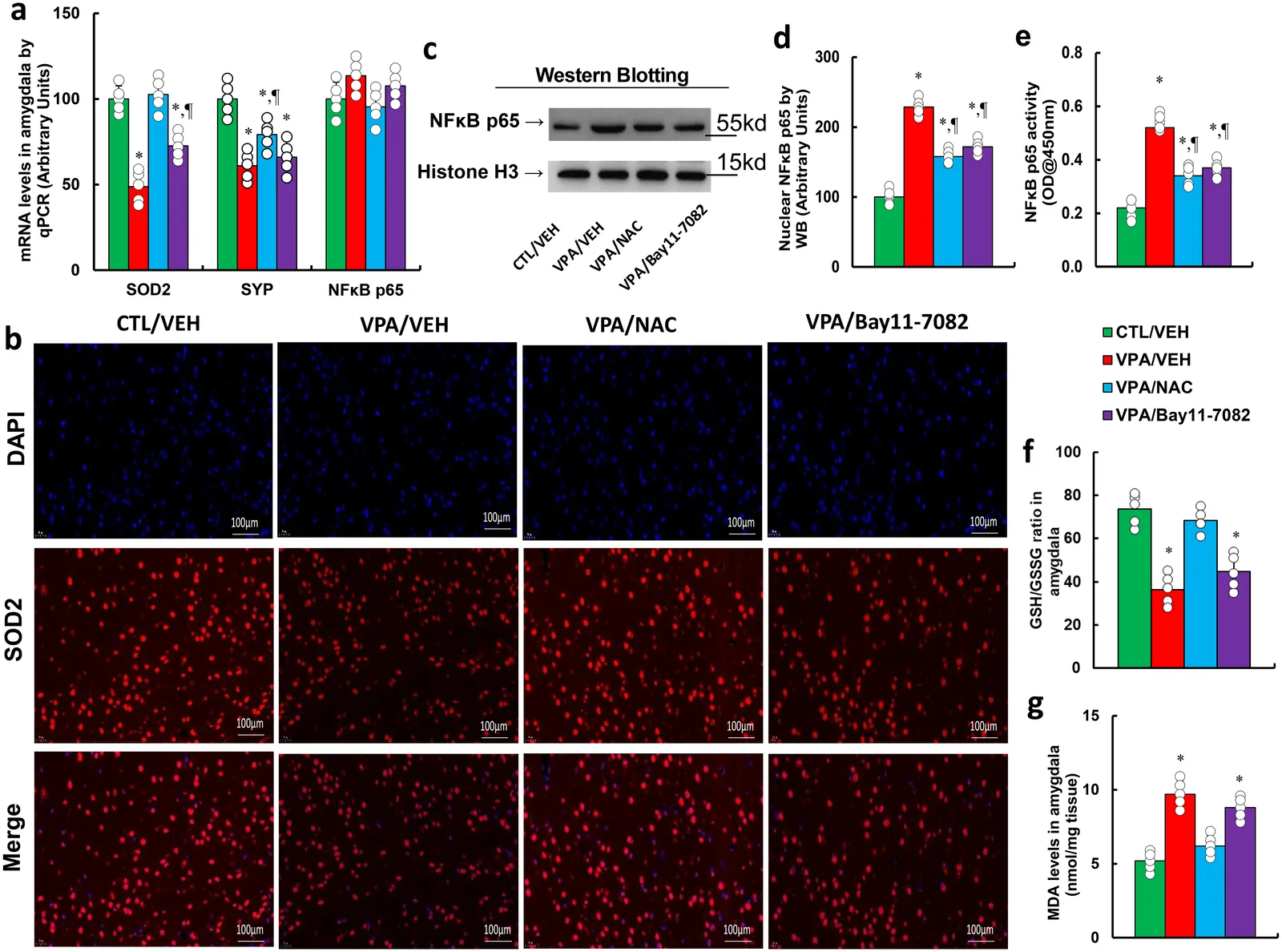
Prenatal VPA-induced oxidative stress and NFκB activation is partly ameliorated by antioxidant NAC, while NFκB inhibitor Bay 11–7082 has minimal effect. Male offspring from CTL or VPA-treated dams received VEH, NAC, or Bay 11-7082. The amygdala tissues were isolated for biological assays. **(a)** mRNA expression. **(b)** Representative IHC for SOD2. **(c)** Representative western blot of nuclear NFκB p65. **(d)** Quantification of **(c)**. **(e)** NFκB p65 activity. **(f)** GSH/GSSG ratio. **(g)** and MDA levels. n=5. *P<0.05 vs. CTL/VEH; ^¶^P<0.05 vs. VPA/VEH. Data are mean ± SEM.

**Figure 8:**
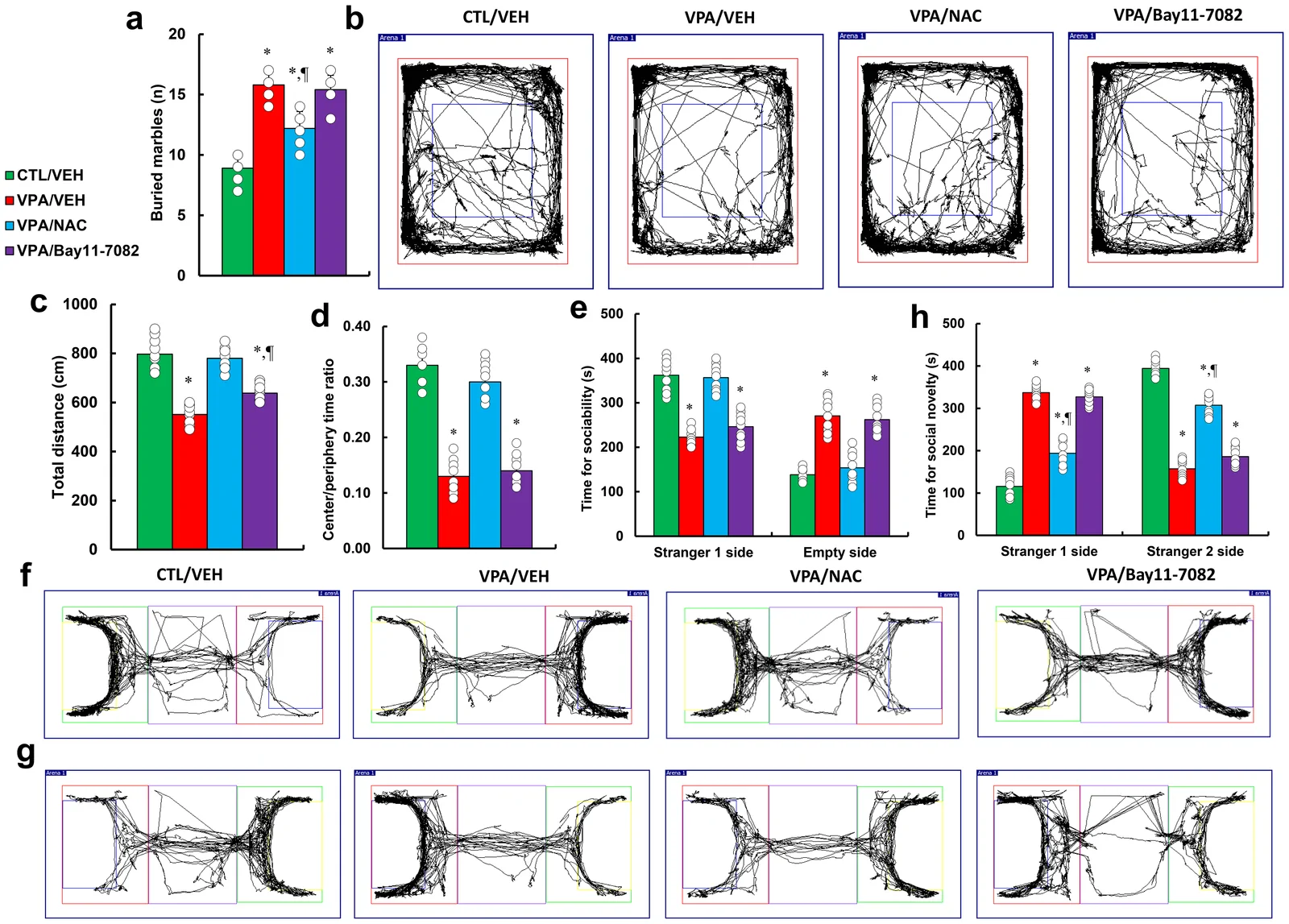
Prenatal VPA-induced autism-like behaviors is partly ameliorated by antioxidant NAC, while NFκB inhibitor Bay 11–7082 has minimal effect. Male offspring from CTL or VPA-treated dams received VEH, NAC, or Bay 11-7082. Behavioral tests were performed. **(a)** marble burying test. **(b–d)** Open-Field Test; **(b)** representative behavioral trajectories; **(c)** total distance traveled; **(d)** center/periphery time ratio. **(e–h)** three-chamber social interaction test; **(e)** sociability; **(f)** representative behavioral trajectories for **(e)**; **(g)** social novelty; **(h)** representative behavioral trajectories for **(g)**. n=9. *P<0.05 vs. CTL/VEH; ^¶^P<0.05 vs. VPA/VEH. Data are presented as mean ± SEM.

In the amygdala, VPA reduced SOD2 and synaptophysin (SYP) expression; NAC fully restored SOD2 and partially rescued SYP, while Bay 11–7082 produced only modest recovery, with no change in total NFκB p65 levels (**Figures 7a, b**). Region-specific effects were observed: in the hypothalamus, VPA-induced SOD2 suppression was partially reversed by both NAC and Bay 11-7082, whereas in the hippocampus, only NAC partially restored SOD2, with no rescue of SYP by either treatment (**Supplementary Figure 5a, b**). Despite unchanged total p65 expression, VPA increased NFκB nuclear translocation and transcriptional activity in the amygdala (**Figures 7c–e**; **Supplementary Figure 4d**), which were partially attenuated by both interventions. Oxidative imbalance, reflected by reduced GSH/GSSG ratio and elevated MDA, was completely normalized by NAC but not by Bay 11-7082 (**Figures 7f, g**). Behaviorally, VPA offspring displayed increased repetitive behavior (**Figure 8a**), reduced locomotion and center exploration (**Figures 8b–d**), impaired sociability, and altered social novelty (**Figures 8e–h**); these deficits were partially rescued by NAC, whereas Bay 11–7082 exerted little effect. Collectively, these findings indicate that restoring redox homeostasis is more effective than direct NFκB inhibition in mitigating VPA-induced molecular and behavioral abnormalities.

### Oxidative stress regulates NFκB activation and inflammatory signaling

**Figure 9:**
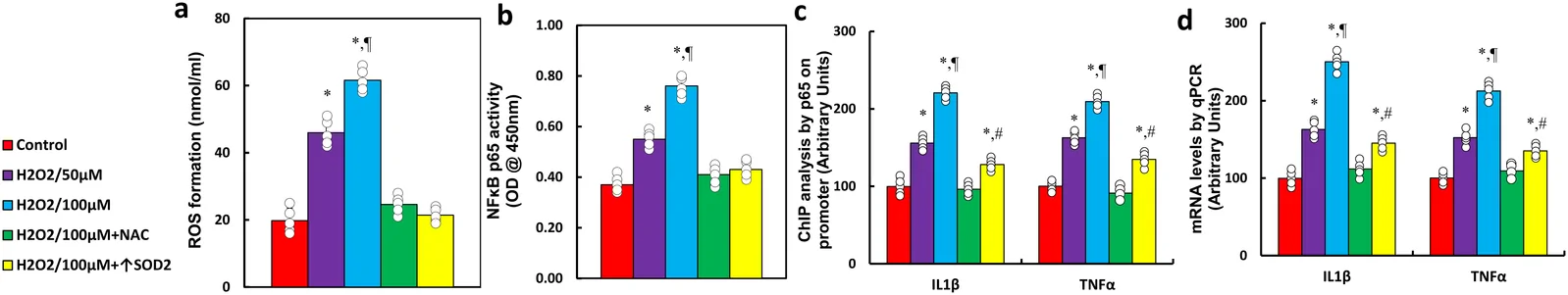
H2O2-induced NFκB activation in primary neurons and rescue by NAC or SOD2 expression. Primary amygdala neurons were treated by H2O2 with either 50 or 100µM in the presence or absence of either 10mM NAC or lentiviral SOD2 overexpression (↑SOD2) for 48 hours, then the neurons were harvested for biological analysis. **(a)** ROS formation. **(b)** NFκB p65 activity assay. **(c)** ChIP analysis by NFκB p65 antibody on promoters. **(d)** mRNA levels for NFκB p65 target genes. n=5. *P<0.05 vs. Control; ^¶^P<0.05 vs. H2O2/50µM; ^#^P<0.05 vs. H2O2/100µM. Data are presented as mean ± SEM.

To investigate the mechanistic relationship between oxidative stress and NFκB signaling, we performed complementary *in vitro* and *in vivo* studies. In primary amygdala neurons, increased oxidative burden elevated intracellular ROS levels and enhanced NFκB p65 activity, which was attenuated by pharmacological antioxidants or genetic enhancement of mitochondrial antioxidant capacity (**Figures 9a, b**). Oxidative stress also increased p65 binding to inflammatory gene promoters (IL1β and TNFα) and elevated their transcript levels, which were reduced following antioxidant interventions (**Figures 9c, d**). In the prenatal VPA model, increased oxidative stress was detected at E18.5, as indicated by elevated MDA levels and reduced GSH/GSSG ratios. These alterations were prevented by prenatal NAC supplementation (**Supplementary Figure 6a, b**). At P7 and P21, VPA-exposed offspring exhibited sustained NFκB p65 activation and increased IL1β expression, which were substantially reduced by prenatal NAC treatment but only partially affected by postnatal intervention (**Supplementary Figure 6c–f**), suggesting that early oxidative stress contributes to persistent NFκB activation. Consistent with these findings, acute pro-oxidant challenge in neonatal mice increased lipid peroxidation, impaired redox homeostasis, and activated NFκB signaling with increased IL1β expression, all of which were attenuated by antioxidant treatment (**Supplementary Figure 7a–d**). Together, these findings support an upstream role of oxidative stress in modulating NFκB activation and inflammatory signaling during ASD-related neurodevelopmental alterations.

### Proposed schematic model of oxidative stress-associated NFκB activation in prenatal VPA-induced autism-like phenotypes

**Figure 10:**
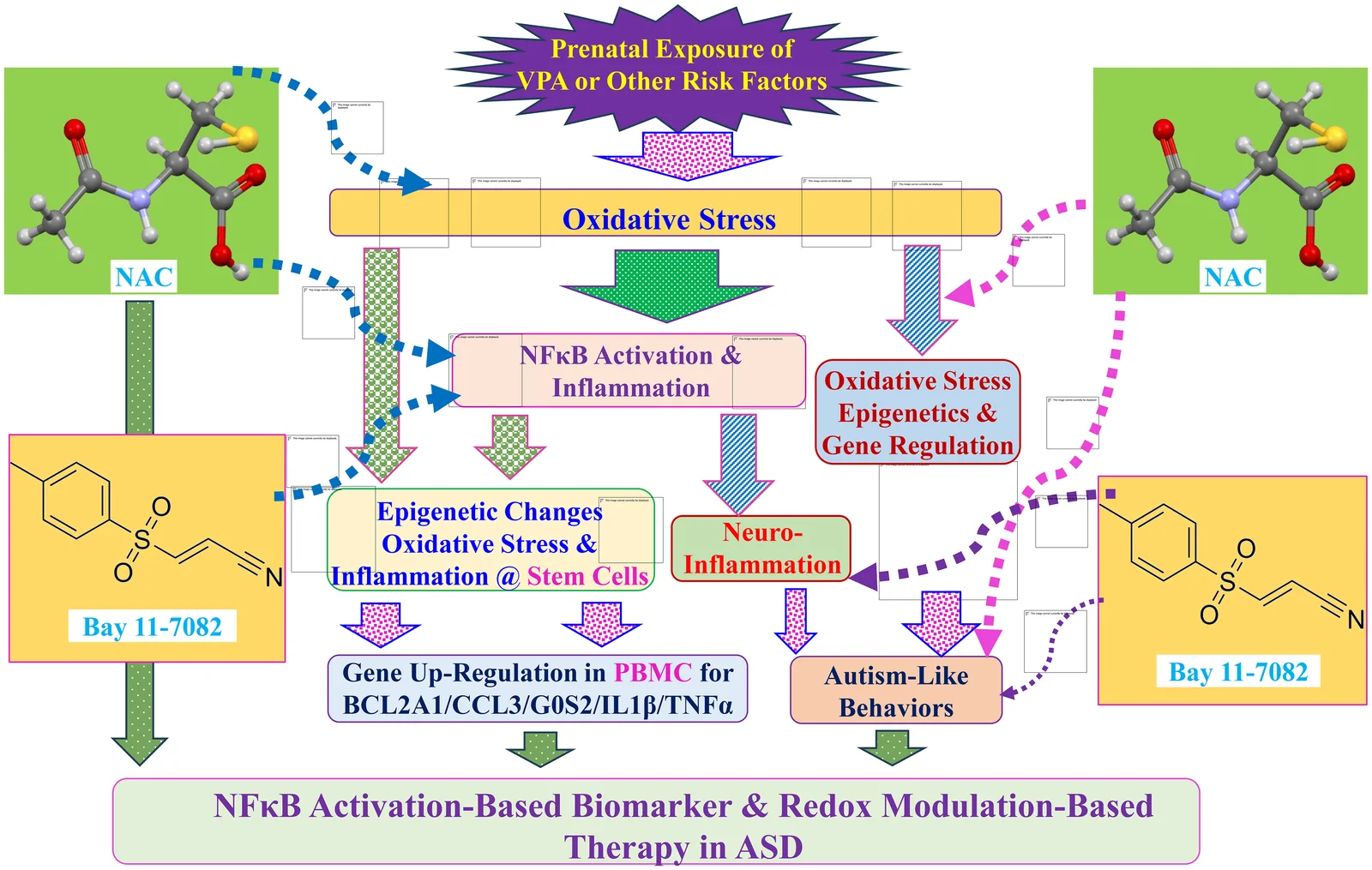
Schematic model of prenatal VPA–induced oxidative stress driving NFκB activation and autism-like phenotypes. ASD, autism spectrum disorder; BCL2A1, BCL2 Related Protein A1; CCL3, C-C Motif Chemokine Ligand 3; G0S2, G0/G1 Switch 2; IL1β, interleukin-1β; NAC, N-acetylcysteine; PBMC, peripheral blood mononuclear cells; TNFα, tumor necrosis factor-α; VPA, valproic acid.

Prenatal exposure to VPA and other potential risk factors is associated with increased oxidative stress. In the brain, redox imbalance is accompanied by NFκB pathway activation, altered inflammatory gene expression, and neuroinflammatory responses associated with ASD-like behavioral abnormalities. In the peripheral system, oxidative stress-associated NFκB activation is accompanied by increased expression of inflammatory and stress-responsive genes, including BCL2A1, CCL3, G0S2, IL1β, and TNFα, in hematopoietic stem cells and PBMCs (4, 40). Treatment with the antioxidant NAC reduces oxidative stress and attenuates NFκB activation, whereas the NFκB p65 inhibitor Bay 11–7082 primarily suppresses NFκB activation with limited effects on oxidative stress markers. Consistent with these molecular changes, NAC partially improves prenatal VPA-induced ASD-like behavioral abnormalities, whereas Bay 11–7082 shows limited behavioral effects. The proposed relationship among oxidative stress, NFκB activation, inflammatory responses, and behavioral phenotypes is illustrated in **Figure 10**.

## Discussion

In this study, we identified persistent oxidative stress and NFκB activation in umbilical cord and peripheral blood from individuals with ASD. In the prenatal VPA mouse model, antioxidant treatment with N-acetyl-L-cysteine (NAC) restored redox homeostasis, reduced NFκB activation, and improved ASD-like behaviors, whereas pharmacological inhibition of NFκB attenuated inflammatory signaling without correcting oxidative imbalance or behavioral deficits. Complementary *in vivo* and *in vitro* experiments further supported that oxidative stress functions upstream of NFκB activation and contributes to sustained neuroinflammatory responses. Collectively, these findings support a model in which oxidative stress is an upstream pathogenic mechanism associated with persistent NFκB-mediated neuroinflammation, while ASD-related behavioral abnormalities likely involve additional oxidative stress-dependent pathways beyond NFκB signaling alone.

### Persistent oxidative stress in ASD-related phenotypes

In our cohort, autistic subjects showed a sustained peripheral redox imbalance from birth through early childhood -lower plasma GSH/GSSG alongside higher MDA and 8-oxo-dG formation -in both umbilical cord and peripheral blood (ages 2–6) versus TD controls, indicating persistent oxidative stress outside the central nervous system. Concordantly, autism-like offspring in the valproate model displayed comparable redox abnormalities in neonatal blood and again at postnatal day 50, aligning with prior reports that VPA exposure elevates peripheral lipid peroxidation and oxidative DNA damage (e.g., MDA↑, 8-oxo-dG↑) and that antioxidant interventions can partially normalize behavior and redox indices (41). These patterns echo meta-analytic evidence: across blood studies in autistic children, GSSG and MDA are increased while the GSH/GSSG ratio is decreased, supporting a systemic shift toward oxidation (42, 43). Notably, oxidative stress signatures extend to the brain -postmortem tissue shows reduced GSH/GSSG and elevated 8-oxo-dG, linking peripheral redox status with central oxidative injury (44). Clinically, urinary 8-OHdG also tracks oxidative DNA damage and may serve as a practical biomarker in ASD (45). Mechanistically, we demonstrated that prenatal metabolic stressors can imprint long-lasting oxidative phenotypes. In maternal diabetes models, hyperglycemia induces persistent ROS production through epigenetic suppression of SOD2, whereas restoring SOD2 activity alleviates redox stress and rescues autism-like behaviors (12). Finally, we recently found that heightened oxidative stress is associated with elevated peripheral BDNF levels in ASD. Moreover, in an autism-like mouse model, restoring systemic redox balance through endothelial or hematopoietic SOD2 augmentation reduced BDNF levels and improved behavioral outcomes, supporting a causal link between chronic oxidative stress, altered neurotrophic signaling, and symptom expression (7). Although 8-oxo-dG is commonly used as a marker of oxidative DNA damage, its predominant cytoplasmic localization may reflect oxidative injury to mitochondrial nucleic acids, including mitochondrial DNA, which is highly susceptible to ROS-induced damage. Potential recognition of oxidized RNA by some antibodies may also contribute to cytoplasmic staining. Thus, the observed 8-oxo-dG signal is consistent with increased mitochondrial oxidative stress in our study. Increasing evidence suggests that oxidative stress-related alterations in ASD may arise from interactions between environmental exposures and genetic susceptibility. Polymorphisms in genes involved in antioxidant defense, mitochondrial function, and redox regulation may influence individual vulnerability to oxidative imbalance following environmental insults. Thus, early-life oxidative stress may represent a convergent pathway through which diverse genetic and environmental risk factors contribute to ASD-related neurodevelopmental abnormalities. Future studies integrating genetic profiling with redox biomarkers will be important to further define these gene–environment interactions (46, 47).

### Persistent NFκB pathway activation in ASD

Our RNA-Seq analysis revealed 16 upregulated genes in umbilical cord blood of ASD subjects compared with typically developing controls, and this elevated expression persisted in peripheral blood between ages 2 and 6 years. All of these genes are directly or indirectly regulated by the NFκB pathway, and five genes with particularly strong upregulation (BCL2A1, CCL3, G0S2, IL1β, and TNFα; fold change > 2.0, ASD/TD ratio > 1.6) were validated by mRNA analysis, suggesting a sustained upstream driving force across embryonic and postnatal development. Consistently, these five genes were also upregulated in the valproic acid (VPA)-induced autism-like mouse model, both in neonatal blood and in peripheral blood at postnatal day 50, accompanied by elevated nuclear NFκB p65 translocation in both human ASD samples and VPA-exposed mice. These findings are consistent with previous reports of systemic NFκB activation in ASD, including increased NFκB DNA-binding activity in PBMC of affected children (19), abnormal NFκB p65 expression and nuclear translocation in postmortem brain tissues (48), and converging evidence from both human and animal studies implicating NFκB signaling in ASD pathophysiology (49). Together, these findings indicate that NFκB pathway activation is persistent and may represent a central mechanism contributing to neuroinflammation and immune dysregulation in ASD.

### Temporal and mechanistic evidence for oxidative stress–mediated NFκB activation

Growing evidence supports oxidative stress as an upstream pathological driver that initiates neuroinflammatory signaling rather than a passive byproduct of inflammation. Excess ROS activate redox-sensitive pathways, including NFκB and MAPK, thereby promoting microglial activation and upregulation of pro-inflammatory cytokines. The resulting neuroinflammatory response further amplifies oxidative injury through increased production of ROS and reactive nitrogen species, establishing a self-reinforcing feed-forward loop that accelerates neuronal dysfunction and behavioral impairment. Experimental studies have demonstrated that oxidative stress precedes and mechanistically drives neuroinflammatory cascades in diverse neurodegenerative and neurodevelopmental models (50, 51). Consistent with these observations, our findings provide strong temporal and mechanistic evidence that oxidative stress acts upstream of NFκB activation in ASD. In umbilical cord and peripheral blood from ASD subjects, elevated oxidative stress was associated with increased expression of NFκB-regulated genes (*BCL2A1, CCL3, G0S2, IL1β*, and *TNFα*), enhanced p65 activity and nuclear translocation (19). VPA-exposed mouse offspring exhibited a similar pattern, with oxidative damage preceding NFκB activation and inflammatory gene upregulation, supporting a directional rather than purely correlative relationship (52). Prenatal antioxidant intervention with NAC attenuated oxidative stress, reduced NFκB activation, and partially rescued behavioral deficits, whereas postnatal treatment was less effective. In contrast, the NFκB inhibitor Bay 11-7082 suppressed pathway activation but did not correct oxidative imbalance or behavioral abnormalities, highlighting the upstream role of redox dysregulation. Complementary in vitro studies in primary neurons confirmed that oxidative stress alone is sufficient to induce p65 nuclear translocation and inflammatory gene transcription, effects reversed by antioxidant treatment or SOD2 overexpression. ChIP assays further demonstrated increased p65 occupancy at promoters of inflammatory genes under oxidative conditions, which was abolished by antioxidant intervention (53). These results collectively support a redox-to-NFκB signaling axis driving neuroinflammation and ASD-like behavioral phenotypes. This aligns with prior reports linking oxidative stress to neuroinflammation and mitochondrial dysfunction in ASD (43, 54), as well as evidence that oxidatively modified cell-free DNA can activate NFκB in ASD lymphocytes (53), and that NAC or other Nrf2-activating antioxidants can restore redox balance and improve behaviors in rodent models (55). Together, these findings reinforce that oxidative stress is not merely a byproduct but a potential pathogenic driver of NFκB activation and behavioral abnormalities, highlighting antioxidant strategies as a rational therapeutic avenue (56). Definitive causality could be further explored using redox-insensitive NFκB mutants, which represents an important direction for future studies.

### NFκB signaling contributes to neuroinflammation but is not the sole driver of ASD-associated behavioral deficits

Although Bay 11–7082 effectively suppressed NFκB activation and reduced neuroinflammatory markers, it failed to substantially improve ASD-like behaviors, indicating that NFκB-mediated inflammation is an important but insufficient contributor to ASD pathophysiology. This result should be interpreted cautiously, as the lack of behavioral rescue may reflect limitations of pharmacological inhibition, including off-target effects or incomplete inhibition in relevant brain regions. Consistent with previous studies, NFκB dysregulation contributes to ASD-associated neuroinflammation, including glial activation and inflammatory signaling, but likely acts within a broader pathological network rather than as a single causal mechanism (48, 57). Prenatal VPA exposure induces multiple interconnected abnormalities, including oxidative stress, mitochondrial dysfunction, synaptic alterations, neurotransmitter imbalance, epigenetic dysregulation, and activation of other redox-sensitive pathways such as mTOR and MAPK. Notably, Bay 11–7082 reduced inflammation without substantially correcting oxidative stress, whereas NAC improved both redox balance and NFκB activation and produced broader behavioral benefits. Together, these findings support oxidative stress as an upstream driver that activates NFκB while also disrupting additional pathogenic pathways, consistent with evidence that NFκB interacts with multiple molecular networks involved in immune regulation, oxidative stress, and neurodevelopment (24). Future genetic studies will be needed to define the specific contribution of individual pathways to ASD-associated behaviors (58).

### Translational considerations and therapeutic implications of redox modulation in ASD

Although our data support a mechanistic link between oxidative stress and NFκB-driven neuroinflammation, the translational implications remain preliminary. For example, while antioxidant intervention such as N-acetylcysteine (NAC) has shown some behavioral benefit in ASD models and early human work (59) the effect on core symptoms remains modest and no pharmacokinetic or rigorous long-term safety evaluations have been reported. Thus, our findings should be viewed as evidence of mechanistic feasibility rather than definitive therapeutic efficacy. The results suggest that restoring redox balance can mitigate NFκB activation and associated neurobehavioral outcomes in the prenatal VPA model, but further studies in additional ASD models- with systematic dose–response, pharmacokinetic, and long-term safety assessments-are warranted (60). Moreover, targeting endogenous antioxidant pathways -such as Nrf2 activation -may offer broader or more sustainable benefits compared with direct NFκB inhibition (52). Overall, these findings refine the translational scope of our work, positioning redox modulation as a promising mechanistic target that warrants deeper preclinical validation before clinical extrapolation.

### Clinical translation outlook

Although our findings identify oxidative stress-driven NFκB activation as a potential contributor to ASD-related neuroinflammation, translation into clinical intervention requires careful consideration. Clinical studies of antioxidant therapies, including NAC supplementation, have shown variable benefits in ASD, suggesting that antioxidant treatment may be effective only in specific subgroups or when administered within appropriate biological windows. Potential limitations include ASD heterogeneity, delayed diagnosis after critical neurodevelopmental periods, insufficient brain availability of some antioxidants, and differences between animal models and human disease. Future translational approaches may therefore require early identification of individuals with redox imbalance through biomarker-based screening, followed by personalized intervention strategies. In addition, targeting endogenous antioxidant pathways, such as activation of the Nrf2 signaling pathway, may provide an alternative strategy to enhance intrinsic antioxidant capacity and overcome some pharmacokinetic limitations of direct antioxidant supplementation. Nutritional supplements with antioxidant properties have also been reported to reduce ROS accumulation and enhance endogenous antioxidant gene expression in experimental models, providing additional support for the feasibility of dietary antioxidant strategies targeting oxidative stress-related disorders. Further longitudinal studies integrating molecular biomarkers, developmental trajectories, and therapeutic responses will be essential to determine whether redox-based interventions can provide clinically meaningful benefits in ASD (61–63).

### Limitations

Several limitations should be considered. First, although our human cohorts identified oxidative imbalance and NFκB activation in ASD, these observational data cannot establish causality. While comprehensive demographic and clinical characteristics, including age, sex, gestational age, birth weight, parental age, family history, cognitive and behavioral assessments, and medication exposure, were collected and analyzed (**Supplementary Tables 2**–**S4**), several potentially relevant variables, such as maternal prenatal antioxidant intake, maternal vitamin D status, children’s dietary habits, selective eating behaviors, gastrointestinal comorbidities, and nutritional interventions, were not systematically collected because the cohorts were established before these factors were incorporated into the study design. Consequently, these variables could not be included in multivariable analyses, and the observed associations between oxidative imbalance and NFκB activation should be interpreted with appropriate caution. Future prospective studies with more comprehensive clinical, dietary, and metabolic characterization are warranted to further evaluate these potential confounding factors and strengthen causal inference (64). Second, peripheral blood and umbilical cord blood biomarkers reflect systemic alterations but may not fully represent CNS-specific processes because of the blood–brain barrier. Although NFκB activation and inflammatory gene expression were increased, microglial activation, CNS inflammation, and protein-level cytokine changes were not directly assessed, and bulk PBMC RNA-seq could not resolve cell-type-specific gene expression or immune cell composition. Future studies using independent cohorts, comprehensive inflammatory profiling, and single-cell or spatial transcriptomic approaches will better define the cellular sources of NFκB activation. Third, our mechanistic findings are based on the prenatal VPA model, which represents only one environmentally induced form of ASD and does not capture the genetic and clinical heterogeneity of the disorder. Although oxidative stress and NFκB dysregulation have also been reported in other ASD models, including BTBR, Shank3 knockout, and maternal immune activation models, their underlying mechanisms may differ; thus, our findings should be interpreted within the context of the VPA model and may apply primarily to a subset of ASD cases (65). Moreover, only male offspring were included because this model produces more robust ASD-like phenotypes in males; therefore, potential sex-specific differences in oxidative stress and NFκB signaling remain to be determined. Future studies incorporating both male and female animals will be important to better understand sex-dependent mechanisms underlying ASD. Finally, although NAC improved oxidative stress, NFκB signaling, and behavioral abnormalities, whereas Bay 11–7082 attenuated inflammation without fully rescuing behavior, both compounds have pharmacological limitations, including the limited blood–brain barrier permeability of NAC and potential off-target effects of Bay 11-7082 (32, 33). In addition, the mechanistic role of NFκB was evaluated primarily using pharmacological inhibition rather than genetic manipulation. Future studies employing cell type-specific or conditional genetic models targeting NFκB pathway components will further strengthen the causal relationship between oxidative stress, NFκB activation, and ASD pathogenesis. Together, these findings indicate that NFκB is an important downstream mediator of oxidative stress but not the sole driver of ASD pathophysiology, which likely involves additional mechanisms such as mitochondrial dysfunction, synaptic abnormalities, and epigenetic dysregulation.

## Conclusions

This study provides converging evidence from longitudinal human cohorts, a prenatal VPA mouse model, and complementary mechanistic experiments that oxidative stress is an upstream event associated with persistent NFκB activation in ASD. Antioxidant intervention with N-acetyl-L-cysteine (NAC) restored redox homeostasis, suppressed NFκB activation, and improved ASD-like behavioral abnormalities, whereas direct inhibition of NFκB attenuated inflammatory signaling without correcting oxidative imbalance or behavioral deficits. These findings support a model in which oxidative stress acts upstream of NFκB-mediated neuroinflammation, with NFκB functioning as a downstream mediator rather than the primary determinant of ASD-related behavioral phenotypes. Collectively, our results highlight redox dysregulation as a potential therapeutic target and provide a mechanistic rationale for investigating early antioxidant strategies to mitigate neuroinflammation and improve neurodevelopmental outcomes in ASD.

## Data Availability

The raw data supporting the conclusions of this article will be made available by the authors, without undue reservation.
