# Supplementary material for: Early-life oxidative stress programs persistent NFκB activation and neuroinflammation in autism spectrum disorder
Source: Front Immunol. 2026 Aug 10;17:1869130. doi: 10.3389/fimmu.2026.1869130 (PMC13500302; doi:10.3389/fimmu.2026.1869130)
Supplement: Supplementary file 1 [file DataSheet1.pdf]

Early-Life Oxidative Stress Programs Persistent NFκB Activation and Neuroinflammation in Autism Spectrum Disorder

Supplemental Information

Data S1. MATERIALS AND METHODS

Reagents and Materials. Antibody for SOD2 (sc-30080) was purchased from Santa Cruz Biotechnology. Antibodies for NFκB p65 (#8242) and Histone H3 (#9715) were obtained from Cell Signaling Technology. Antibody for CCL3 (ab214819) was obtained from Abcam. Protein concentration was measured using the Coomassie Protein Assay Kit (Pierce Biotechnology). The NE-PER Nuclear and Cytoplasmic Extraction Kit (#78835) was purchased from Thermo Scientific, China. The 8-oxo-dG antibody (4354-MC-050) was obtained from Novus Biologicals. Menadione (#M5625), N-Acetyl-L-cysteine (NAC, #A7250), NFκB inhibitor BAY 11-7082 (#196870), rotenone (#557368) and valproic acid (VPA, #P4543) were purchased from Sigma, China. NFκB p65 activity was measured by TransAM® NFκB p65 Chemi Kit (#40597, from Active Motif) per manufacturers' instructions.

Human study protocol. The human study was carried out in accordance with the recommendations of the Human Subject Guidelines of the Human Subjects Institutional Review Board. The protocol was approved by Hainan Women and Children's Medical Center, and all subjects gave written informed consent in accordance with the Declaration of Helsinki.

Human longitudinal follow-up study. A longitudinal follow-up study was conducted at Hainan Women and Children's Medical Center, China, enrolling all newborns delivered between July 2018 and July 2019. Umbilical cord blood (5–20 mL) was collected at birth into EDTA-containing tubes, and umbilical cord blood mononuclear cells (UCBMCs) were isolated. Total RNA was extracted using the RNeasy Micro Kit (Qiagen) and stored for downstream analyses, including RNA-seq and gene-specific assays. Clinical and demographic information, including parental age, pregnancy and birth characteristics, family history, and other perinatal factors, was collected at enrollment using standardized questionnaires. All children underwent developmental and behavioral assessments every 6 months from birth to 3 years of age (July 2019–July 2022). Autism screening was performed using the Autism Behavior Checklist (ABC) and Autism Spectrum Quotient (AQ). ASD diagnoses were confirmed according to DSM-5 criteria by licensed clinical psychologists and psychiatrists. Cognitive and adaptive functioning were evaluated using the Mullen Scales of Early Learning (MSEL) and the Vineland Adaptive Behavior Scales (VABS). Information regarding participant age, sex, developmental status, and medication use, when available, was recorded and reviewed during follow-up assessments. Typically developing (TD) children were defined by the absence of ASD or developmental delay and MSEL/VABS scores >70 (1, 2). Based on these evaluations, 29 children were diagnosed with ASD and 31 age- and sex-comparable children were classified as TD (see Table S2). UCBMC samples from these individuals were used for RNA-seq analysis (Fig. S1a) (2). In addition, an independent validation cohort of ASD and TD children (n = 41) was recruited from a separate population, and plasma and UCBMC samples were collected for validation studies (see Table S3, Fig. S1b).

Population-based case-control study. A population-based case-control study was conducted involving children aged 2–6 years after obtaining written informed consent from their parents or legal guardians. ASD diagnoses (n = 30) were established using the Autism Behavior Checklist and confirmed by a multidisciplinary clinical team according to DSM-5 criteria. Typically

developing (TD) controls (n = 28) were randomly selected from a pool of 2,519 TD children and matched to ASD participants by age, sex, and cognitive performance, as assessed by the Wechsler Intelligence Scale for Children-IV (WISC-IV). Demographic and clinical information, including age, sex, developmental assessments, and medication status when available, was collected for all participants. TD participants had no history of ASD, intellectual disability, or developmental delay and scored >70 on both MSEL and VABS assessments (see details in Table S2). Peripheral blood samples (3–5 mL) were collected and processed to obtain plasma and peripheral blood mononuclear cells (PBMCs). Plasma was used to measure oxidative stress markers, including the GSH/GSSG ratio and malondialdehyde (MDA) levels (Fig. S1a) (1, 2). PBMCs were used for RNA-seq, quantitative real-time PCR, chromatin immunoprecipitation (ChIP), and 8-oxo-dG immunostaining analyses. An independent validation cohort of ASD and TD participants (n = 56) was additionally recruited from a separate population, and plasma and PBMC samples were collected for confirmatory analyses (see Table S4, Fig. S1b) (2).

RNA Sequencing and Bioinformatic Analysis. Total RNA was extracted from samples using TRIzol reagent (Invitrogen, USA) according to the manufacturer's instructions. RNA purity and concentration were assessed with a NanoDrop 2000 (Thermo Scientific, USA), and integrity was verified using the Agilent 2100 Bioanalyzer (Agilent, USA). RNA-seq libraries were prepared with the VAHTS Universal V6 RNA-seq Library Prep Kit and sequenced using the Illumina NovaSeq 6000 platform to generate 150 bp paired-end reads (OE Biotech Co., Ltd., Shanghai, China). Raw sequencing reads were quality-controlled and filtered using fastp (3). Clean reads were aligned to the reference genome with HISAT2 (4). Gene-level counts were quantified using HTSeq (5) and normalized as fragments per kilobase per million (FPKM). Differential gene expression analysis was performed using DESeq2 with cutoffs of $|\log_2 \text{fold change}| > 1$ and adjusted $p < 0.05$ (6). Principal component analysis (PCA) and hierarchical clustering were conducted in *R* (v3.2.0) to evaluate sample variability. Enrichment analysis of Gene Ontology (GO), KEGG pathways, Reactome, and WikiPathways was conducted using a hypergeometric test in *R*. Gene Set Enrichment Analysis (GSEA) was carried out with the GSEA software (7) to identify functionally enriched gene sets. Additional analyses included transcript assembly with StringTie (8), alternative splicing detection via ASprofile, SNP/InDel calling using SAMtools and BCFtools, and variant effect prediction using SnpEff.

Preparation of SOD2 expression lentivirus. The cDNA for mouse SOD2 (obtained from Open Biosystems) was subcloned into the pLVX-Puro vector (from Clontech) with the underlined restriction sites using the following primers: SOD2 forward primer: 5'- gtac- ctcgag- atg ttg tgt cgg gcg gcg tgc-3' (Xho1) and SOD2 reverse primer: 5'- gtac- tctaga- tca ctt ctt gca agc tgt gta -3' (Xba1). The lentivirus for SOD2 or empty control (CTL) was expressed through Lenti-X™ Lentiviral Expression Systems (from Clontech) per manufacturers' instructions, and the prepared lentivirus was used for infection of cells for the expression of either empty (EMP) or SOD2 (↑SOD2).

RT reaction and real-time quantitative PCR. Total RNA from treated cells was extracted using the RNeasy Micro Kit (Qiagen), and the RNA was reverse transcribed using an Omniscript RT kit (Qiagen). All the primers were designed using Primer 3 Plus software with the Tm at 60°C, primer size of 21 bp, and product length in the range of 140-160 bp (see Table S1). The primers were validated with an amplification efficiency in the range of 1.9-2.1 and the amplified products were confirmed with agarose gel. Real-time quantitative PCR was run on iCycler iQ (Bio-Rad) using the Quantitect SYBR green PCR kit (Qiagen). The PCR was performed by denaturing at 95°C for 8min followed by 45 cycles of denaturation at 95°C, annealing at 60°C, and extension at 72°C for 10s, respectively. 1µl of each cDNA was used to measure target genes. β-actin was used as the housekeeping gene for transcript normalization, and the mean values were used to calculate

relative transcript levels with the $^{\Delta\Delta}$CT method per instructions from Qiagen. In brief, the amplified transcripts were quantified by the comparative threshold cycle method using β-actin as a normalizer. Fold changes in gene mRNA expression were calculated as $2^{-\Delta\Delta CT}$ with CT = threshold cycle, ΔCT=CT (target gene)-CT(β-actin), and the ΔΔCT =ΔCT (experimental)-ΔCT (reference).

Chromatin immunoprecipitation (ChIP). Cells were washed and crosslinked using 1% formaldehyde for 20min and terminated by 0.1M glycine. Cell lysates were sonicated and centrifuged. 500µg of protein were pre-cleared by BSA/salmon sperm DNA with preimmune IgG and a slurry of Protein A Agarose beads. Immunoprecipitations were performed with the indicated antibodies, BSA/salmon sperm DNA and a 50% slurry of Protein A agarose beads. Input and immunoprecipitates were washed and eluted before then being incubated with 0.2mg/ml Proteinase K for 2h at 42˚C, followed by 6h at 65˚C to reverse the formaldehyde crosslinking. DNA fragments were recovered through phenol/chloroform extraction and ethanol precipitation. A ~150 bp fragment on the related promoter was amplified by real-time PCR (qPCR) using the primers provided in Table S1.

Measurement of oxidative stress. ROS production was quantified using CM-H2DCFDA (10µM; Invitrogen) incubated at 37°C for 45min. Fluorescence was determined at 485/530nm and quantified as nmol/mL using an H2O2 calibration curve. The 8-oxo-dG formation was determined by immunostaining and quantitated by Image J. The 8-OHdG in plasma was measured by Human 8-OHdG ELISA Kit (# CEA660Ge) from Cloud-Clone Corp (Wuhan, China). The GSH/GSSG ratio was measured using the GSH/GSSG-Glo™ Assay Kit (#V6611, obtained from Promega) per manufacturers' instructions.

Measurement of Malondialdehyde (MDA). MDA concentrations in plasma and tissue samples were quantified using a commercial ELISA kit (Nanjing Jiancheng Bioengineering Institute, China) according to the manufacturer's instructions. Plasma was collected into anticoagulant-treated tubes, centrifuged at 2,000×g for 10 min at 4°C, and the supernatant stored at −80°C until analysis. For tissues, samples were weighed, homogenized in ice-cold phosphate-buffered saline (PBS; pH 7.4) at a ratio of 1:9 (w/v), and centrifuged at 10,000 × g for 10 min at 4°C; the resulting supernatants were stored at −80°C. Prior to assay, samples were thawed on ice and loaded into wells precoated with MDA-specific antibodies, alongside a series of MDA standards to generate a standard curve. After incubation and washing, horseradish peroxidase (HRP)-conjugated secondary antibody was added, followed by the chromogenic substrate 3,3',5,5'-tetramethylbenzidine (TMB). The reaction was stopped with 2 mol/L sulfuric acid, and absorbance was read at 450 nm using a microplate reader. MDA concentrations were calculated from the standard curve and expressed as nmol/mL for plasma or nmol/mg protein for tissues.

Immunostaining. The treated cells were transferred to cover slips coated with 0.1% gelatin, fixed by 3.7% formaldehyde at 37ºC for 15 min, permeabilized by 1% BSA+0.2% Triton X-100 in PBS for 1 hour, and then blotted with 40µg/ml (dilute 1:50) of related antibody for CCL3, NFκB p65 or 8-oxo-dG for 2 hours. The cells were then washed three times and the FITC/Texas Red labeled anti-mouse/rabbit secondary antibody (1:100) was added for blotting for another 1 hour. After thorough washing, the slides were visualized and photographed; cell nuclei were stained with 4',6-diamidino-2-phenylindole dihydrochloride (DAPI, #D9542, from Sigma) and the staining was quantitated by Image J (9).

In Vivo Animal Experiments. All animal procedures were conducted in accordance with the U.S. National Institutes of Health guidelines (Guide for the Care and Use of Laboratory Animals, No. 85-23, revised 1996) and were reviewed and approved by the Institutional Animal Care and Use Committee of Hainan Women and Children's Medical Center (#2020006). Experimental

C57BL/6J mice were housed in a specific pathogen–free facility maintained at 22 ± 2 °C with relative humidity of 50 ± 10% under a 12:12-h light–dark cycle, with ad libitum access to standard rodent chow and water. Ten timed-pregnant dams were assigned to each prenatal exposure group. From each litter, one male offspring was randomly selected for subsequent experiments to ensure statistical independence and to control for litter effects. To account for potential perinatal mortality, at least nine viable male offspring per group were included in the final analyses. After weaning, each selected offspring was housed individually in separate cages to minimize potential confounding effects of maternal care or social interactions.

Postnatal Treatment on Prenatal VPA Exposure-Mediated Autism-Like Offspring. To investigate the therapeutic effects of postnatal NFκB inhibition or antioxidant intervention in a prenatal VPA-induced autism model, timed-pregnant C57BL/6 mice were administered a single intraperitoneal injection of valproic acid (VPA; 600mg/kg, dissolved in sterile saline) on gestational day (GD) 12.5, while control dams received an equal volume of saline. After birth, only male offspring were selected and randomly assigned into four groups: Group 1: CTL/VEH (prenatal saline + postnatal vehicle), Group 2: VPA/VEH (prenatal VPA + postnatal vehicle), Group 3: VPA/NAC (prenatal VPA + postnatal NAC), and Group 4: VPA/Bay11-7082 (prenatal VPA + postnatal Bay11-7082). From postnatal day (PND) 21 to 41, mice received once-daily intraperitoneal injections of either vehicle (0.5% DMSO in PBS), NAC (150 mg/kg), or Bay11-7082 (5mg/kg). Animals were maintained under standard housing conditions with ad libitum access to food and water. The neonatal PBMC was isolated during the PND of 1-5. Behavioral assessments were conducted between PND 42 and 49. On PND 50, animals were euthanized, and blood, plasma and peripheral blood mononuclear cells (PBMC) were isolated. Brain tissues were dissected, snap-frozen for molecular analysis, or fixed for immunohistochemistry. Gene expression was assessed by qPCR, NFκB pathway activation was evaluated by western blot. Levels of proinflammatory cytokines in plasma were measured by ELISA. Oxidative stress markers, including ROS, malondialdehyde (MDA) and GSH/GSSG ratio were quantified to evaluate redox imbalance (see Fig S2).

Animal behavior tests. Behavioral assessments were conducted in mouse offspring using the Marble-Burying Test (MBT) to evaluate repetitive behavior, the Open-Field Test to assess locomotor activity and anxiety-like behavior, and the Three-Chambered Social Test to measure sociability and social novelty preference. All tests were recorded with a ceiling-mounted camera, and raw behavioral trajectories were analyzed using automated tracking software (SMART) by investigators blinded to treatment groups. For the Open-Field Test, total distance traveled and time spent in center versus periphery were quantified, while in the Three-Chambered Social Test, time spent in each chamber and social preference ratios were calculated. Representative trajectory plots were presented alongside summary statistics. Autism-like phenotypes were operationally defined as increased repetitive behaviors, heightened anxiety-like responses, or reduced sociability and impaired social novelty preference relative to control mice (9-13).

Marble-Burying Test (MBT). Repetitive and compulsive-like behaviors were assessed using the marble-burying test. Each mouse was individually placed in a clean polypropylene cage (35 × 23 × 19 cm^3) containing 5 cm of fresh wood-chip bedding. Twenty colored glass marbles (1 cm diameter) were evenly arranged in a 5 × 4 grid on the bedding surface. Mice were allowed to explore the cage freely for 30 min under standard room lighting conditions. At the end of the test, a marble was considered buried when more than 50% of its surface was covered by bedding material. The total number of buried marbles was recorded as an index of repetitive behavior. To minimize observer bias, scoring was performed independently by two investigators blinded to the experimental groups, and the average value was used for statistical analysis. The testing

apparatus was cleaned and fresh bedding was provided between animals to eliminate olfactory cues (10-12).

Open-Field Test (OFT). General locomotor activity and anxiety-like behavior were assessed using the open-field test. Each mouse was gently placed in the center of a square open-field arena (50 × 50 × 40 cm) and allowed to explore freely for 10 min under approximately 100 lux illumination. Animal movements were continuously recorded using a ceiling-mounted digital camera connected to an automated video-tracking system. The arena was virtually divided into a central zone (25 × 25 cm) and a peripheral zone. Total distance traveled (cm) was quantified as a measure of locomotor activity, while time spent in the center zone (s) and the number of center entries were used as indices of anxiety-like behavior. Increased peripheral exploration and reduced center activity were interpreted as elevated anxiety-like behavior. The arena was thoroughly cleaned with 70% ethanol and allowed to dry between trials to remove residual odors. All behavioral testing and data analysis were performed by investigators blinded to treatment allocation (14).

Three-Chambered Social Test. Sociability and social novelty were assessed using a three-chamber apparatus (each chamber: ~20 × 40 × 22 cm) in mouse offspring. Target mice ("Stranger 1" and "Stranger 2") were 7–8 weeks old and habituated to wire cages for 3 consecutive days prior to testing, while test mice were acclimated to the testing room for at least 45 min. During the sociability phase, the test mouse was placed in the middle chamber for a 5-min habituation, followed by a 10-min session in which Stranger 1 occupied one side-chamber and an empty wire cage was placed in the other. In the social novelty phase, Stranger 2 was introduced into the previously empty cage, and the test mouse was allowed to explore all chambers for 10 min. Behavioral tracking, including time spent in each chamber, number of entries, and raw locomotor trajectories, was recorded using SMART video-tracking software. Reduced time spent in the chamber containing Stranger 1 or Stranger 2, or altered exploration patterns, was defined as deficits in sociability or social novelty preference, respectively. All behavioral scoring and analyses were performed by observers blinded to treatment groups (9, 13).

In vitro primary culture of amygdala neurons. Amygdala tissues were dissected from rats on embryonic day 18 (E18 rats). Tissues were treated with 0.05% trypsin EDTA for 15 min at 37°C. Trypsin EDTA was replaced with soybean trypsin inhibitor (Sigma) for 5 min at 37°C to stop the reaction. This was then replaced with supplemented Neurobasal A (Invitrogen) followed by mechanical dissociation. Cells were then resuspended in culture media, including Neurobasal A, B27, 1×GlutaMAX and 100 U/ml Pen/Strep (from Invitrogen), and then the cells were incubated at 37°C, 5% CO2 (15). The isolated amygdala neurons were used for immunostaining, analysis of DNA methylation, and epigenetic changes by ChIP assay (16).

Preparation of PBMC and plasma from Neonatal Mice via Decapitation. Neonatal mice (postnatal day 1–5) were euthanized by rapid decapitation with sterile surgical scissors. Immediately following decapitation, the body was held vertically over a sterile 1.5 mL low-retention microcentrifuge tube preloaded with 2–5 μL of anticoagulant (0.5 M EDTA) to prevent coagulation. Blood was allowed to drain passively from the neck, and gentle massage from tail to neck was applied to maximize yield. Samples were kept on ice and processed within 1 hour. For plasma preparation, collected blood was centrifuged at 2000×g for 10 minutes at 4°C. The plasma (supernatant) was carefully transferred to fresh RNase-/DNase-free microcentrifuge tubes and stored at −80°C until analysis. To isolate blood cells, the remaining pellet was resuspended in 500 μL of sterile phosphate-buffered saline (PBS) and centrifuged again at 500×g for 5 minutes at 4°C to wash. The supernatant was discarded, and the cell pellet containing PBMC were used immediately for the analysis of gene expression, oxidative stress, and NFκB p65 activation.

Typical yields ranged from 50–100 µL of whole blood per pup, providing approximately 20–50 µL of plasma and sufficient cells for molecular analysis.

Isolation of brain tissues. The brain tissues were isolated from experimental mice for further biological assays. The experimental mice were deeply anesthetized through free breathing of isoflurane vapor (>5%). The whole blood was then withdrawn by heart puncture for PBMC isolation and the mouse was perfused transcardially by 20 ml cold perfusion solution for 5 min. The skull was cut using a pair of small surgical scissors and the brain was carefully freed from the skull before being transferred to a petri dish (60 mm×15 mm) that was filled with ice-cold DPBS solution. The targeted brain regions, including the amygdala, hypothalamus (PVN area) and hippocampus, were dissected under the surgical microscope under the referred location from the atlas outlined in *The Mouse Brain in Stereotaxic Coordinates (3rd Edition)*. A separate petri dish was prepared for each of the target regions. The whole dissection process was carried out in the span of no more than one hour. The dissected tissues were then frozen at -80°C for either immediate use or later biological assays (17, 18).

Immunohistochemistry (IHC). Brain tissues were sectioned into 10µm slices using a cryostat microtome. Sections were fixed in 2% paraformaldehyde for 15 minutes at room temperature, followed by fixation in methanol for 10 minutes at 4°C. After fixation, tissues were permeabilized with 1% bovine serum albumin (BSA) and 0.2% Triton X-100 in phosphate-buffered saline (PBS). Sections were then incubated with anti-SOD2 antibody (40µg/mL) for 2 hours at room temperature. Subsequently, samples were incubated for 1 hour with Texas Red–conjugated secondary antibodies. Finally, sections were mounted with fluorescent mounting medium containing 4',6-diamidino-2-phenylindole (DAPI) and covered with coverslips. Slides were visualized and photographed using a fluorescence microscope, and SOD2 protein expression was quantified in 60 cells per group using ImageJ software (19).

Analysis of proinflammatory cytokines by ELISA. Cytokine levels (BCL2A1, CCL3, G0S2, IL1β and TNFα) were measured in human and mouse plasma, using species-specific ELISA kits (from R&D Systems, China). Samples were centrifuged and stored at −80°C prior to use. Assays were conducted according to the manufacturer's instructions. Briefly, 96-well plates pre-coated with capture antibodies were incubated with standards and diluted samples at 37°C for 2 hours. After washing, biotinylated detection antibodies and HRP-conjugated streptavidin were added sequentially. 3,3′,5,5′-Tetramethylbenzidine (TMB) substrate was applied for color development, followed by 2N H_2SO_4 to stop the reaction. Absorbance was read at 450nm. Cytokine concentrations were calculated using standard curves. All samples were analyzed in duplicate. Human and mouse assays were run in parallel using matched antibody pairs and species-specific standards.

Temporal and Mechanistic Validation of Oxidative Stress–Driven NFκB Activation. For the developmental time-course analysis, amygdala tissues from VPA-exposed and control offspring were collected at embryonic day 18.5 (E18.5), postnatal day 7 (P7), and postnatal day 21 (P21), and analyzed for redox indices (GSH/GSSG ratio and MDA), NFκB activation, and inflammatory gene expression by RT-qPCR (see Fig S3a). To assess sufficiency, wild-type P7 pups were acutely treated with pro-oxidants (rotenone or menadione) and examined 24–48 h later for ROS generation and NFκB activation. To evaluate the temporal requirement for antioxidant intervention, NAC was administered either prenatally to dams (E10.5–E18.5) or postnatally to pups (P7–P21), followed by assessment of redox status and NFκB signaling. Primary amygdala neurons were used to interrogate cellular mechanisms (see Fig S3b). Hydrogen peroxide (H_2O_2) induced ROS production and NFκB p65 activation in a dose-dependent manner, whereas NAC treatment or SOD2 overexpression (↑SOD2) effectively attenuated these responses. ChIP assays were

performed to quantify p65 occupancy at the IL1β and TNFα promoters (see Fig S3c). All experiments included appropriate vehicle controls and technical standards, and analyses were performed in a blinded manner.

Statistics and reproducibility. Data are presented as mean ± SEM. Comparisons between two groups were performed using unpaired two-tailed Student's t-tests. Differences in categorical variables were analyzed using the χ^2 test. For experiments involving three or more groups, statistical significance was assessed using one-way analysis of variance (ANOVA) followed by Tukey–Kramer post hoc tests for multiple pairwise comparisons. The biochemical and molecular biomarkers evaluated by ELISA, qPCR, Western blotting, and related assays were selected based on a priori hypotheses derived from the RNA-seq findings and were analyzed independently. For RNA-seq analyses, differential gene expression was determined using DESeq2, and P values were adjusted for multiple testing using the Benjamini–Hochberg false discovery rate (FDR) procedure. Statistical analyses were performed using SPSS 22.0 and R software. A two-sided P value < 0.05 was considered statistically significant (20).

REFERENCES

1. Zhang X, Fu C, Wang M, Feng D, Wang H, Li H, et al. Brain-derived neurotrophic factor levels and oxidative stress in autism: evidence from children and a mouse model. *J Psychiatry Neurosci.* 2025;50(4):E218-E33.
2. Jia Q, Li H, Wang M, Wei C, Xu L, Ye L, et al. Transcript levels of 4 genes in umbilical cord blood are predictive of later autism development: a longitudinal follow-up study. *J Psychiatry Neurosci.* 2023;48(5):E334-E44.
3. Chen S, Zhou Y, Chen Y, and Gu J. fastp: an ultra-fast all-in-one FASTQ preprocessor. *Bioinformatics.* 2018;34(17):i884-i90.
4. Kim D, Langmead B, and Salzberg SL. HISAT: a fast spliced aligner with low memory requirements. *Nat Methods.* 2015;12(4):357-60.
5. Anders S, Pyl PT, and Huber W. HTSeq--a Python framework to work with high-throughput sequencing data. *Bioinformatics.* 2015;31(2):166-9.
6. Love MI, Huber W, and Anders S. Moderated estimation of fold change and dispersion for RNA-seq data with DESeq2. *Genome Biol.* 2014;15(12):550.
7. Subramanian A, Tamayo P, Mootha VK, Mukherjee S, Ebert BL, Gillette MA, et al. Gene set enrichment analysis: a knowledge-based approach for interpreting genome-wide expression profiles. *Proc Natl Acad Sci U S A.* 2005;102(43):15545-50.
8. Pertea M, Pertea GM, Antonescu CM, Chang TC, Mendell JT, and Salzberg SL. StringTie enables improved reconstruction of a transcriptome from RNA-seq reads. *Nat Biotechnol.* 2015;33(3):290-5.
9. Wang X, Lu J, Xie W, Lu X, Liang Y, Li M, et al. Maternal diabetes induces autism-like behavior by hyperglycemia-mediated persistent oxidative stress and suppression of superoxide dismutase 2. *Proc Natl Acad Sci U S A.* 2019;116(47):23743-52.
10. Bahi A. Hippocampal BDNF overexpression or microR124a silencing reduces anxiety- and autism-like behaviors in rats. *Behav Brain Res.* 2017;326:281-90.
11. Zou Y, Lu Q, Zheng D, Chu Z, Liu Z, Chen H, et al. Prenatal levonorgestrel exposure induces autism-like behavior in offspring through ERβ suppression in the amygdala. *Mol Autism.* 2017;8:46.
12. Xie W, Ge X, Li L, Yao A, Wang X, Li M, et al. Resveratrol ameliorates prenatal progestin exposure-induced autism-like behavior through ERβ activation. *Mol Autism.* 2018;9:43.

13. Moy SS, Nadler JJ, Perez A, Barbaro RP, Johns JM, Magnuson TR, et al. Sociability and preference for social novelty in five inbred strains: an approach to assess autistic-like behavior in mice. *Genes Brain Behav.* 2004;3(5):287-302.
14. Seibenhener ML, and Wooten MC. Use of the Open Field Maze to measure locomotor and anxiety-like behavior in mice. *J Vis Exp.* 2015(96):e52434.
15. Hay CW, Shanley L, Davidson S, Cowie P, Lear M, McGuffin P, et al. Functional effects of polymorphisms on glucocorticoid receptor modulation of human anxiogenic substance-P gene promoter activity in primary amygdala neurones. *Psychoneuroendocrinology.* 2014;47:43-55.
16. Zou Y, Lu Q, Zheng D, Chu Z, Liu Z, Chen H, et al. Prenatal levonorgestrel exposure induces autism-like behavior in offspring through ERbeta suppression in the amygdala. *Mol Autism.* 2017;8:46.
17. Liu L, Zhou X, and Wu JY. Preparing Viable Hippocampal Slices from Adult Mice for the Study of Sharp Wave-ripples. *Bio Protoc.* 2020;10(19):e3771.
18. Liu L, Besson-Girard S, Ji H, Gehring K, Bulut B, Kaya T, et al. Dissociation of microdissected mouse brain tissue for artifact free single-cell RNA sequencing. *STAR Protoc.* 2021;2(2):100590.
19. Zhang H, Li L, Chen Q, Li M, Feng J, Sun Y, et al. PGC1beta regulates multiple myeloma tumor growth through LDHA-mediated glycolytic metabolism. *Mol Oncol.* 2018;12(9):1579-95.
20. Kim DH, Krakowiak P, Meltzer A, Hertz-Picciotto I, and Van de Water J. Neonatal chemokine markers predict subsequent diagnosis of autism spectrum disorder and delayed development. *Brain Behav Immun.* 2022;100:121-33.

Table S1. Sequences of primers for the real time quantitative PCR (qPCR)

Gene	Species	Analysis	Forward primer (5'→3')	Reverse primer (5'→3')
β-actin	Human	mRNA	gatgcagaaggagatcactgc	atactcctgcttgctgatcca
BCL2A1	Human	mRNA	cgtagacactgccagaacact	tatccacatccggggcaattt
CCL3	Human	mRNA	ccttgctgtcctcctctgc	ggctgctcgtctcaaagtagt
G0S2	Human	mRNA	gtggtgctcggcctgatg	ctgcttgcctttctcctgca
IL1β	Human	mRNA	ctgaaagctctccacctccag	tttcaacacgcaggacaggta
NFκB p65	Human	mRNA	agcttgtaggaaaggactgcc	ggttgttgttggtctggatgc
TNFα	Human	mRNA	cctgtagcccatgttgtagca	aggacctgggagtagatgagg
CCL3	Human	ChIP	ggatatcctgagcccctgtg	gtctgaaaccagctctcctct
β-actin	Mouse	mRNA	tcttgggtatggaatcctgtg	atctccttctgcatcctgtca
BCL2A1	Mouse	mRNA	aactggggaaggattgtgact	gccgtatccattctcctgtgt
CCL3	Mouse	mRNA	ccaagtcttctcagcgccata	gccggtttctcttagtcagga
G0S2	Mouse	mRNA	cctagttgagacggtgtgcag	gtcgcctcctgtgccttg
IL1β	Mouse	mRNA	gcccatcctctgtgactcat	tccattgaggtggagagctt
NFκB p65	Mouse	mRNA	cctcatctttccctcagagcc	tggtatctgtgcttctctcgc
SOD2	Mouse	mRNA	ggcctacgtgaacaatctcaa	tcaggtttgtccagaaaatgg
SYP	Mouse	mRNA	ttcgctttcatgtggctagtt	aagtcacagggtccctcagtt
TNFα	Mouse	mRNA	ctcacaaaccaccaagtgga	ccttgtcccttgaagagaacc
TNFα	Mouse	ChIP	acacacacacaccctcctgat	agtggccctacacctctgtct
IL1β	Mouse	ChIP	gaagtgcgtgtctctccagaa	acgtagatgcacacccagaag
CCL3	Mouse	ChIP	tcttagatatcctgggcccct	gccccttctcttataggtagcc

Table S2. Demographic and clinical characteristics of ASD and TD participants

Characteristics	Longitudinal follow-up cohort		Population-based case-control cohort	
	ASD (n=29)	TD (n=31)	ASD (n=30)	TD (n=28)
Age at assessment	36.2 ± 1.8 months	36.5 ± 1.7 months	4.1 ± 1.0 years	4.0 ± 1.1 years
Male sex, n (%)	24 (82.8%)	25 (80.6%)	25 (83.3%)	23 (82.1%)
Gestational age (weeks)	39.1 ± 1.2	39.0 ± 1.1	N/A	N/A
Birth weight (g)	3,250 ± 380	3,290 ± 360	N/A	N/A
Parental age (years)	30.4 ± 3.8	30.1 ± 3.6	31.0 ± 4.2	30.7 ± 4.0
Family history of neurodevelopmental disorders	2 (6.9%)	1 (3.2%)	2 (6.7%)	1 (3.6%)
ABC score	52.4 ± 12.6	7.8 ± 4.1	55.1 ± 13.8	8.2 ± 4.5
AQ score	28.6 ± 6.5	12.4 ± 4.3	30.1 ± 7.2	11.9 ± 4.0
MSEL total score	61.8 ± 8.9	92.4 ± 10.2	64.2 ± 9.7	94.1 ± 11.0
VABS adaptive behavior score	65.3 ± 9.6	96.7 ± 10.8	68.1 ± 10.4	98.2 ± 11.5
IQ (WISC-IV)	N/A	N/A	76.5 ± 10.2	82.1 ± 9.8
Medication use at blood collection, n (%)	0 (0%)	0 (0%)	2 (6.7%)	0 (0%)
Antipsychotic/psychotropic medication exposure	0 (0%)	0 (0%)	0 (0%)	0 (0%)

Note: Data are presented as mean ± SEM or n (%). Abbreviations: ASD, autism spectrum disorder; TD, typically developing; ABC, Autism Behavior Checklist; AQ, Autism Spectrum Quotient; MSEL, Mullen Scales of Early Learning; VABS, Vineland Adaptive Behavior Scales; WISC-IV, Wechsler Intelligence Scale for Children-IV.

Table S3. Demographic and clinical characteristics of the independent validation cohort from the longitudinal follow-up study.

Characteristics	ASD (n=41)	TD (n=41)	P value
Age at assessment (months)	36.1 ± 2.0	36.3 ± 1.8	0.65
Male sex, n (%)	33 (80.5%)	33 (80.5%)	>0.99
Gestational age (weeks)	39.0 ± 1.1	39.2 ± 1.0	0.42
Birth weight (g)	3,245 ± 365	3,295 ± 350	0.53
Parental age (years)	30.6 ± 3.9	30.2 ± 3.7	0.64
Family history of neurodevelopmental disorders, n (%)	3 (7.3%)	2 (4.9%)	0.64
ABC score	51.7 ± 12.4	7.6 ± 4.0	<0.001
AQ score	28.4 ± 6.3	12.1 ± 4.2	<0.001
MSEL total score	62.5 ± 9.2	93.1 ± 10.3	<0.001
VABS adaptive behavior score	66.8 ± 9.9	97.5 ± 10.9	<0.001
Medication use, n (%)	1 (2.4%)	0 (0%)	0.31

Note: Data are presented as mean ± SEM or n (%). ASD diagnoses were confirmed according to DSM-5 criteria. TD participants showed no evidence of ASD or developmental delay and had MSEL and VABS scores >70.

Table S4. Demographic and clinical characteristics of the independent validation cohort from the population-based case-control study.

Characteristics	ASD (n=56)	TD (n=56)	P value
Age (years)	4.1 ± 1.0	4.0 ± 1.0	0.72
Male sex, n (%)	46 (82.1%)	45 (80.4%)	0.82
Parental age (years)	31.1 ± 4.2	30.8 ± 4.0	0.71
Family history of neurodevelopmental disorders, n (%)	4 (7.1%)	2 (3.6%)	0.40
ABC score	55.6 ± 13.1	8.1 ± 4.3	<0.001
AQ score	30.2 ± 6.9	11.8 ± 3.9	<0.001
WISC-IV IQ score	77.8 ± 10.5	82.9 ± 9.6	0.06
VABS adaptive behavior score	68.7 ± 10.3	98.4 ± 11.2	<0.001
Medication use, n (%)	3 (5.4%)	0 (0%)	0.08
Psychotropic medication use, n (%)	0 (0%)	0 (0%)	N/A

Note: Data are presented as mean ± SEM or n (%). ASD diagnoses were confirmed according to DSM-5 criteria. TD participants were selected from the same source population and matched as closely as possible for age and sex.

FIGURE S1

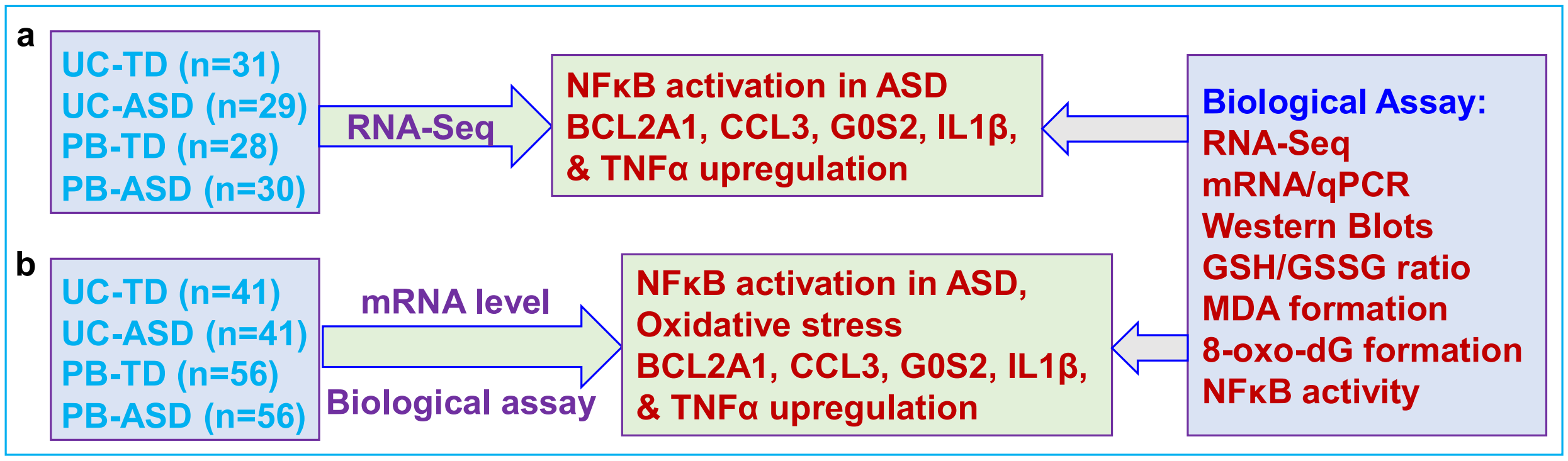

Figure S1. Human study protocol for umbilical cord (UC) and peripheral blood (PB) sample collection. (a) Human blood samples used for RNA sequencing (RNA-seq). (b) Human blood samples used for mRNA expression analysis and other biological assays. Abbreviations: ASD, autism spectrum disorders; BCL2A1, BCL2 Related Protein A1; CCL3, C-C Motif Chemokine Ligand 3; G0S2, G0/G1 Switch 2; IL1β, interleukin 1β; MDA, malondialdehyde; NFκB, Nuclear Factor kappa-light-chain-enhancer of activated B cells; PB, peripheral blood; TD, typically developing; TNFα, Tumor Necrosis Factor α. UC, umbilical cord.

FIGURE S2

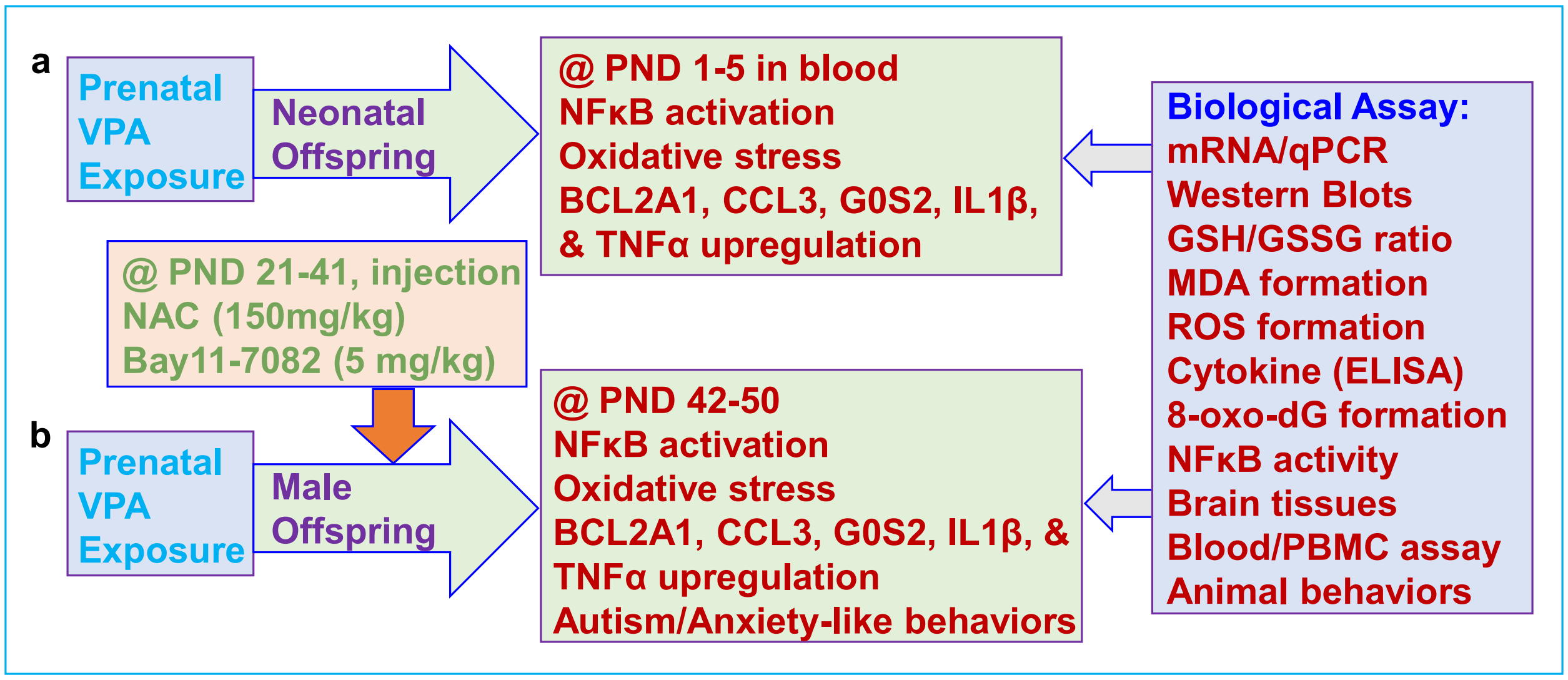

Figure S2. Protocol for prenatal VPA-induced behavioral deficits and therapeutic interventions with the antioxidant NAC and the NFκB inhibitor Bay 11-7082. (a) Experimental design to evaluate the effects of prenatal VPA exposure in neonatal offspring. (b) Experimental design to evaluate the effects of NAC and Bay 11-7082 treatment on behavioral deficits in male offspring prenatally exposed to VPA. Abbreviations: BCL2A1, BCL2 Related Protein A1; CCL3, C-C Motif Chemokine Ligand 3; G0S2, G0/G1 Switch 2; IL1β, interleukin 1β; MDA, malondialdehyde; NAC, N-acetylcysteine; NFκB, Nuclear Factor kappa-light-chain-enhancer of activated B cells; PND, postnatal day; PBMC, peripheral blood mononuclear cells; ROS, reactive oxygen species; TNFα, Tumor Necrosis Factor α; VPA, valproic acid.

FIGURE S3

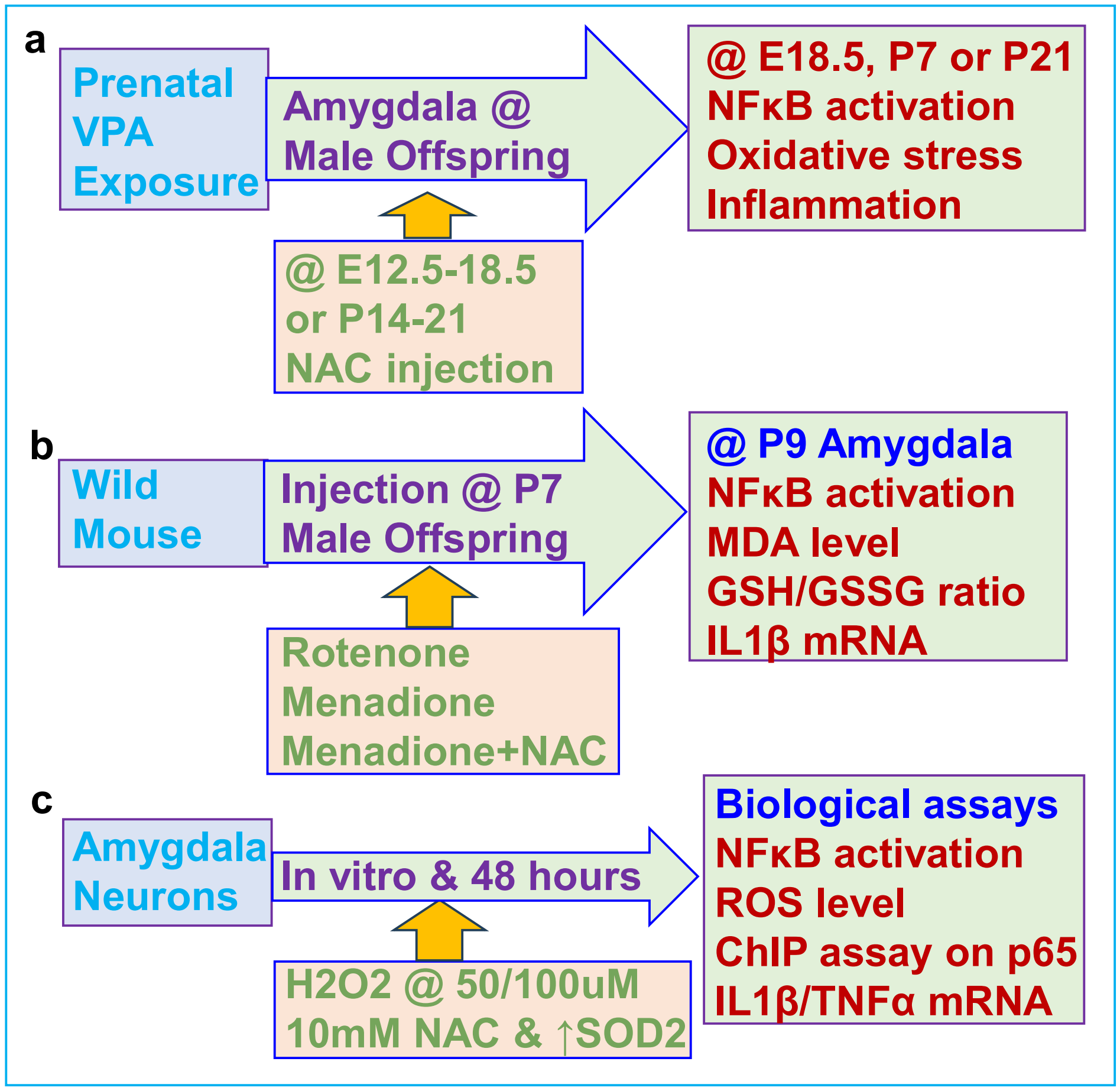

Figure S3. In vivo and in vitro experimental protocols for temporal and mechanistic validation of oxidative stress–driven NFκB activation. (a) Experimental design to evaluate the effects of prenatal and postnatal NAC treatment on NFκB activation following prenatal VPA exposure. (b) Experimental design to assess the effects of postnatal pro-oxidant treatment on NFκB activation. (c) In vitro dose–response protocol for H_2O_2-induced NFκB activation. Abbreviations: ASD, autism spectrum disorders; E, embryonic; P, postnatal; ChIP, chromatin immunoprecipitation; G0S2, G0/G1 Switch 2; IL1β, interleukin 1β; MDA, malondialdehyde; NAC, N-acetylcysteine; NFκB, Nuclear Factor kappa-light-chain-enhancer of activated B cells; ROS, reactive oxygen species; SOD2, superoxide dismutase 2; TNFα, Tumor Necrosis Factor α; VPA, valproic acid.

FIGURE S4

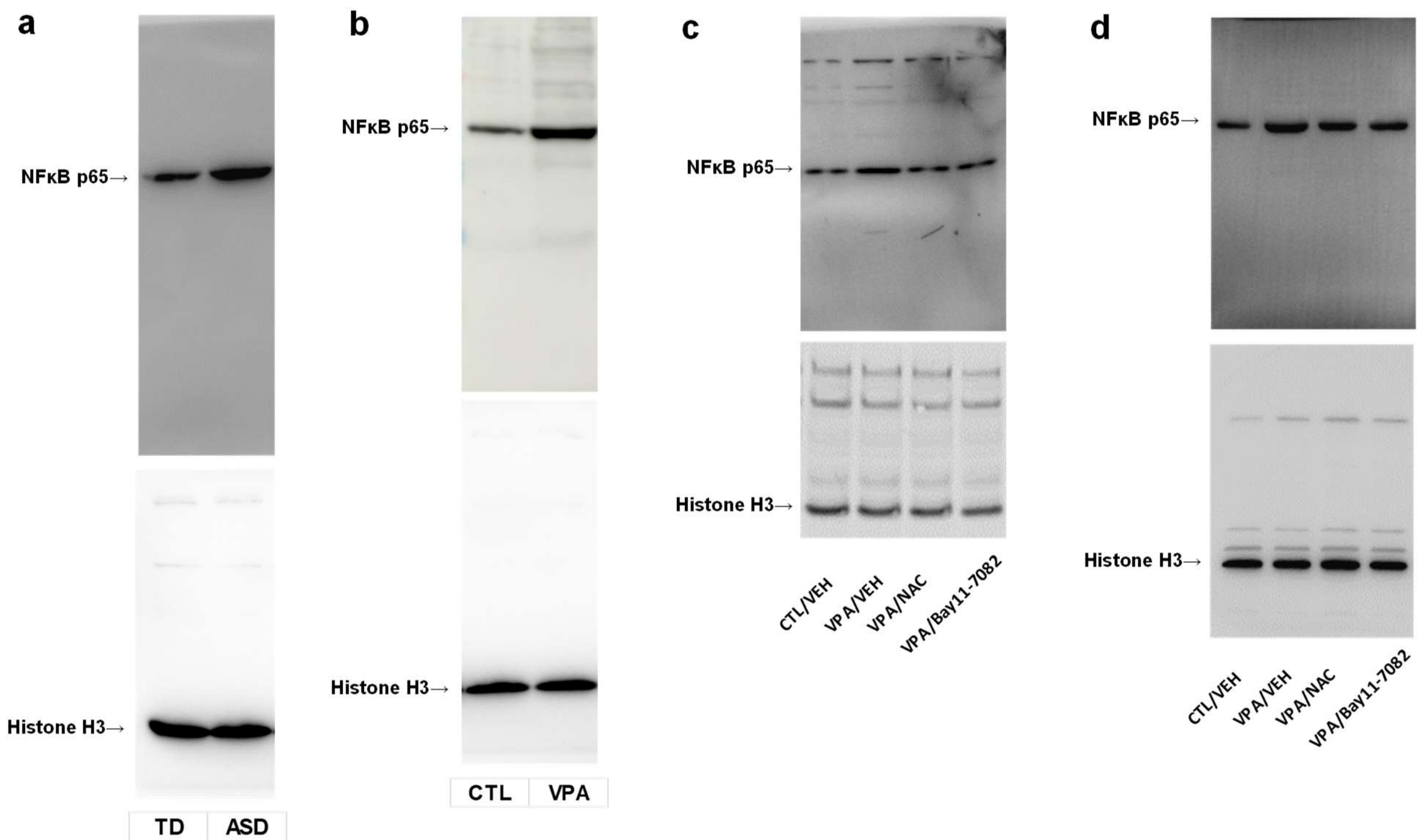

Figure S4. Representative pictures of full blots for Western Blotting. (a) Full blots for Figure 2k. (b) Full blots for Figure 4g. (c) Full blots for Figure 6f. (d) Full blots for Figure 7c.

FIGURE S5

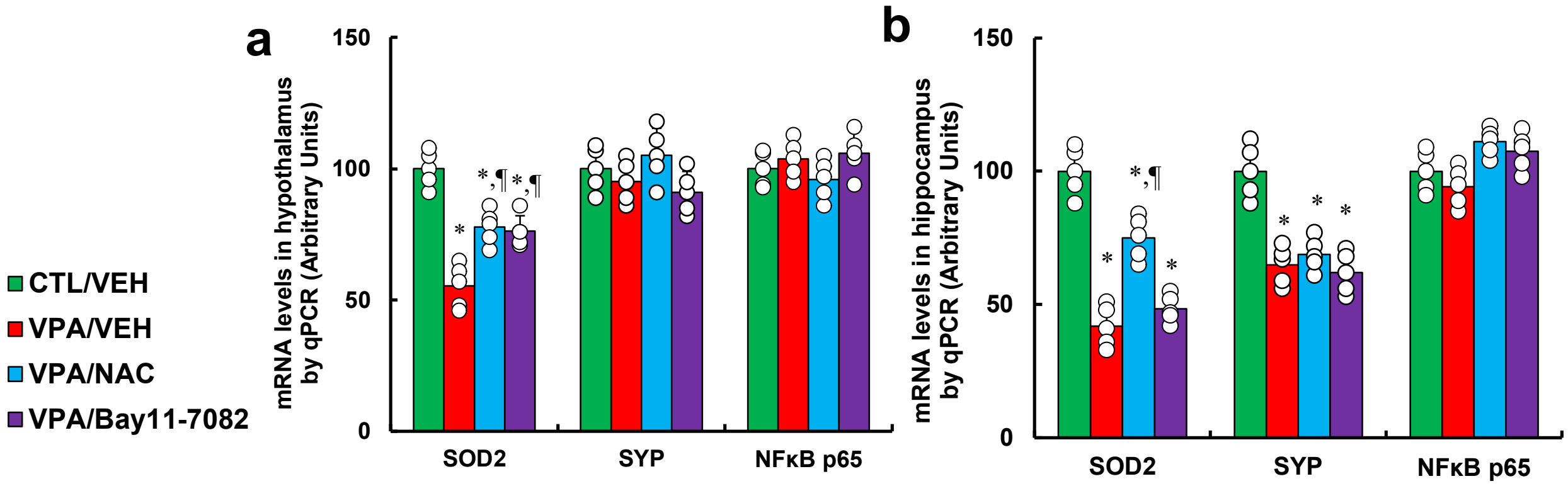

Figure S5. Prenatal VPA exposure induced- altered gene expression in brain is partly ameliorated by antioxidant NAC and NFκB inhibitor Bay 11-7082. Male offspring from control (CTL) or VPA-treated dams received injection of vehicle (VEH), NAC (150 mg/kg), or Bay11-7082 (5 mg/kg), and the brain tissues were isolated for biological assays. (a) mRNA levels in hypothalamus. (b) mRNA levels in hippocampus. n=5. *,P<0.05 vs. CTL/VEH group; ¶,P<0.05 vs. VPA/VEH group. Data are presented as mean ± SEM.

FIGURE S6

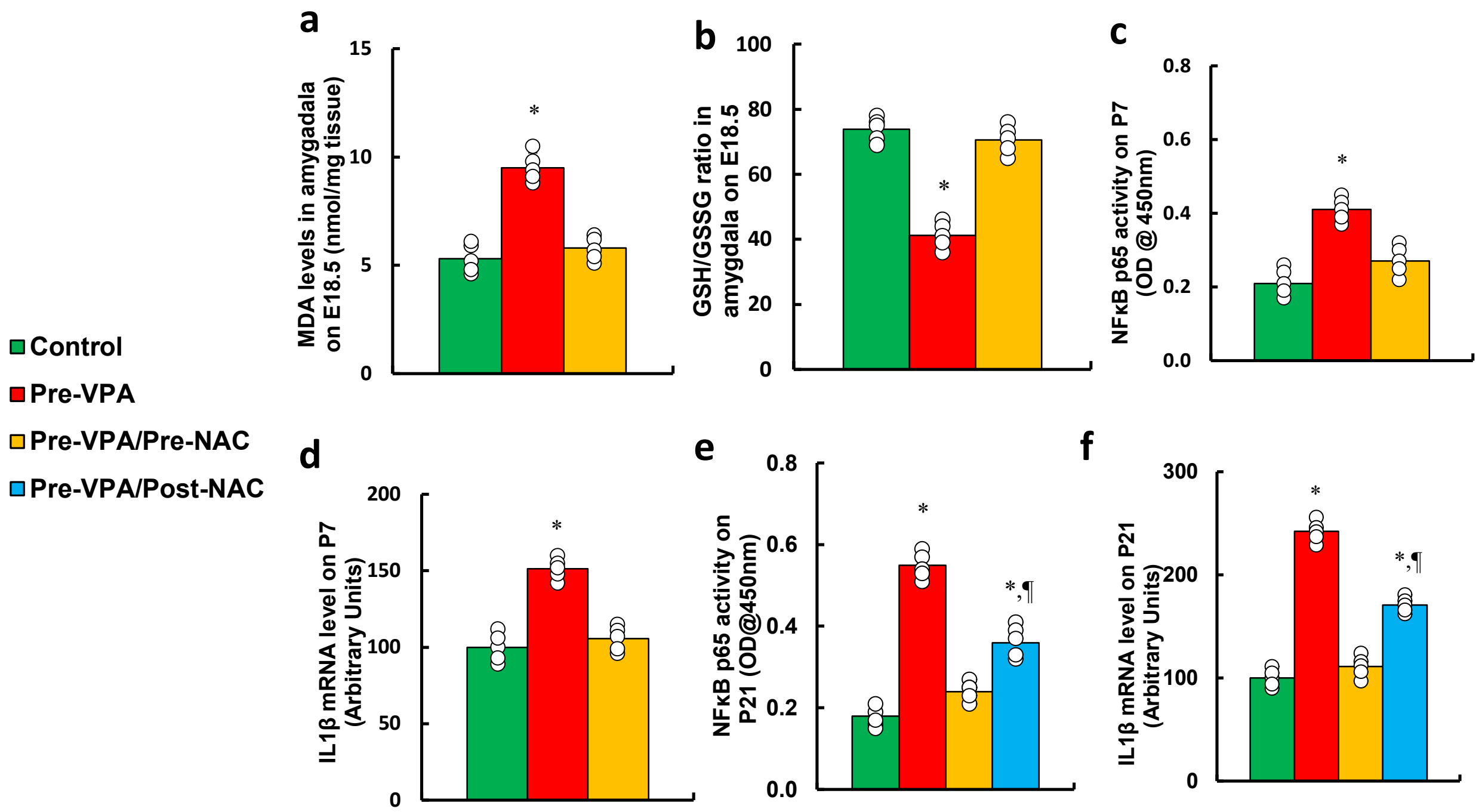

Figure S6. Developmental time-course of redox imbalance and NFκB activation. Pregnant dams were administered by a single intraperitoneal injection of valproic acid (Pre-VPA; 600mg/kg, dissolved in sterile saline) on gestational day (GD) 12.5, while control dams received an equal volume of saline. Some animals with Pre-NAC group received once-daily intraperitoneal injections of either vehicle (0.5% DMSO in PBS) or NAC (150 mg/kg) from E12.5 until baby birth. Also, some animals with Post-NAC group received once-daily intraperitoneal injections of either vehicle (0.5% DMSO in PBS) or NAC (150 mg/kg) from P14-P21. Amygdala tissues were collected from offspring at E18.5, P7 and P21 for biological analysis. (a) MDA levels on E18.5. (b) GSH/GSSG ratio on E18.5. (c) NFκB p65 activity on P7. (d) IL1β mRNA on P7. (e) NFκB p65 activity on P21. (f) IL1β mRNA on P21. n=5. *, $P<0.05$ vs. Control; ¶, $P<0.05$ vs. Pre-VPA. Data are presented as mean ± SEM.

FIGURE S7

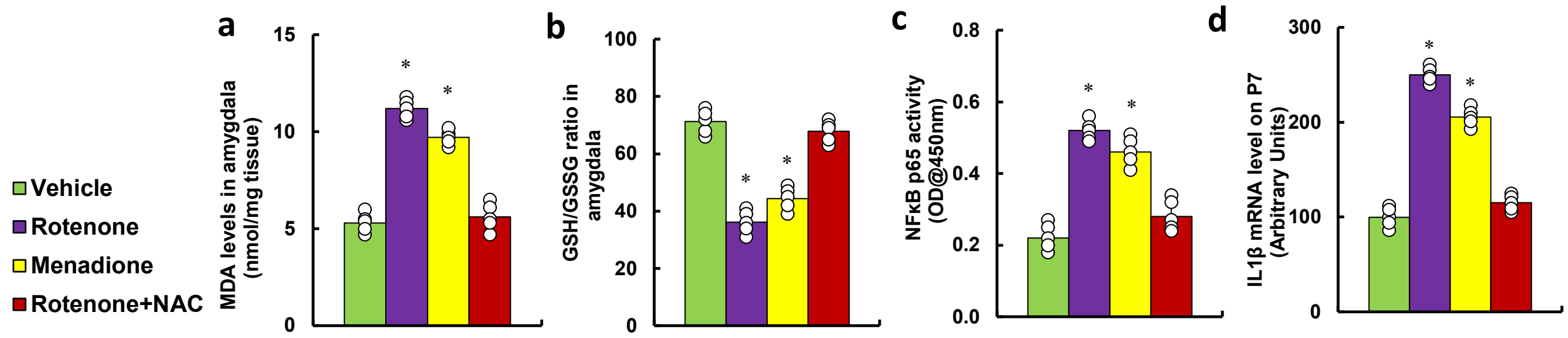

Figure S7. Acute pro-oxidant challenge demonstrates sufficiency of oxidative stress for NFκB p65 activation. Wild-type P7 pups received a single intraperitoneal dose of rotenone (0.5mg/kg) or menadione (10mg/kg) in the presence or absence of NAC (150mg/kg). Amygdala tissues were collected 48 h later for biological analysis. (a) MDA levels. (b) GSH/GSSG ratio. (c) NFκB p65 activity. (d) IL1β mRNA. n=5. *, $P<0.05$ vs. Vehicle. Data are presented as mean ± SEM.
